# A pay for performance scheme in primary care: Meta-synthesis of qualitative studies on the provider experiences of the quality and outcomes framework in the UK

**DOI:** 10.1186/s12875-020-01208-8

**Published:** 2020-07-13

**Authors:** Nagina Khan, David Rudoler, Mary McDiarmid, Stephen Peckham

**Affiliations:** 1 Independent Researcher, Ontario, Canada; 2 Faculty of Health Sciences, University of Ontario Institute of Technology, 2000 Simcoe Street North, Unit UA3000, Oshawa, ON L1H 7K4 Canada; 3 Ontario Shores Centre for Mental Health Sciences, 700 Gordon Street, Whitby, ON L1N 5S9 Canada; 4 Centre for Health Services Studies, University of Kent, Kent, Canterbury CT2 7NF UK

**Keywords:** Pay for performance (P4P), Primary care, Quality and outcomes framework (QOF), Quality initiatives, Qualitative research, Meta-synthesis, Meta-ethnography, Qualitative synthesis

## Abstract

**Background:**

The Quality and Outcomes Framework (QOF) is an incentive scheme for general practice, which was introduced across the UK in 2004. The Quality and Outcomes Framework is one of the biggest pay for performance (P4P) scheme in the world, worth £691 million in 2016/17. We now know that P4P is good at driving some kinds of improvement but not others. In some areas, it also generated moral controversy, which in turn created conflicts of interest for providers. We aimed to undertake a meta-synthesis of 18 qualitative studies of the QOF to identify themes on the impact of the QOF on individual practitioners and other staff.

**Methods:**

We searched 5 electronic databases, Medline, Embase, Healthstar, CINAHL and Web of Science, for qualitative studies of the QOF from the providers’ perspective in primary care, published in UK between 2004 and 2018. Data was analysed using the Schwartz Value Theory as a theoretical framework to analyse the published papers through the conceptual lens of Professionalism. A line of argument synthesis was undertaken to express the synthesis.

**Results:**

We included 18 qualitative studies that where on the providers’ perspective. Four themes were identified; 1) Loss of autonomy, control and ownership; 2) Incentivised conformity; 3) Continuity of care, holism and the caring role of practitioners’ in primary care; and 4) Structural and organisational changes**.** Our synthesis found, the Values that were enhanced by the QOF were power, achievement, conformity, security, and tradition. The findings indicated that P4P schemes should aim to support Values such as benevolence, self-direction, stimulation, hedonism and universalism, which professionals ranked highly and have shown to have positive implications for Professionalism and efficiency of health systems.

**Conclusions:**

Understanding how practitioners experience the complexities of P4P is crucial to designing and delivering schemes to enhance and not compromise the values of professionals. Future P4P schemes should aim to permit professionals with competing high priority values to be part of P4P or other quality improvement initiatives and for them to take on an ‘influencer role’ rather than being ‘responsive agents’. Through understanding the underlying Values and not just explicit concerns of professionals, may ensure higher levels of acceptance and enduring success for P4P schemes.

## Background

Internationally, there has been substantial interest in the use of pay for performance (P4P) schemes for primary care in high, medium and low-income countries. The longest standing and most comprehensive scheme, is the Quality and Outcomes Framework (QOF) for United Kingdom (UK) general practice. However, in the UK there have been increasing calls for the QOF to be abolished and in 2016 Scotland ended the scheme. The QOF now continues only in England, Wales and Northern Ireland [1, 2]. In early 2017, the British Medical Association (BMA) called for the QOF to be suspended to reduce bureaucratic pressures and free up clinical time [3]. In April 2016, National Health Service (NHS) England commenced a review of the QOF, acknowledging that it may have ‘served its purpose’ and may be ‘a barrier to holistic management’ [4, 5]. Published in July 2018 the *Review of the Quality and Outcomes Framework in England* [6], concluded that the scheme should be revised with a greater emphasis on an approach that would “… increase the likelihood of improved patient outcomes, decrease the likelihood of harm from over-treatment and improve personalisation of care” (p11). Among the recommended changes, the report outlined an approach that included supporting practices to undertake quality improvement activities set out in the GP contract for 2019/20 [6]. It also supported the development of pooled incentive schemes or shared savings programs for networks of practices [6]. In England the proposal for shared savings and financial incentive schemes signals a shift from the focus on individual practices with new incentive schemes seeking to influence primary care professional behaviour through more collective and quality improvement approaches to “… *facilitate achievement of system efficiencies and increase income for reinvestment to primary care networks*” [6].

While QOF has predominantly had a clinical practice focus (some process and organisational criteria were dropped after just a few years), it has always had a practice wide impact and studies suggest it has had a significant influence on the functioning and organisation of practices [6, 7]. Though the QOF has had an impact on clinical practice, it has also had some unintended consequences. Understanding the importance and impact of these consequences is useful for decision-makers designing P4P schemes [7].

To date, most studies of the QOF have used quantitative methods to evaluate the impact of QOF on clinical performance [8–10] and the universally high QOF achievement means that practices have little motivation to improve achievement further. However, ‘high performance’ does not necessarily mean ‘high quality’ [6]. Motivation to deliver high quality care among health professionals is complex, but it is likely that other motivational factors other than financial rewards may be effective [6]. Therefore, it is important to consider other ways of motivating health professionals to deliver high quality care [6]. MacDonald and others have argued that it is possible to avoid unintended consequences of P4P systems if they are designed with the involvement of clinicians and aligned with their underlying values [11, 12]. As governments are developing schemes for quality improvement, they need relevant and context-sensitive evidence to support policy interventions, which means that there is significant ambiguity over the optimal design of such schemes to maximise efficiency and tolerability. Decision-makers are increasingly using qualitative evidence to understand various socioeconomic contexts, health systems and communities [13]. Furthermore, this type of evidence is useful to assess the needs, values, perceptions and experiences of stakeholders, including policymakers, providers, communities and patients, and is thus crucial for complex health decision-making [7, 13].

For this paper, we conducted a meta-synthesis of the available qualitative research on QOF to identify lessons that will be useful for decision-makers in designing and implementing new incentive schemes. Drawing on evidence from the UK provides the widest range of studies on one scheme from which to develop clear lessons for those factors that might support or hinder particular behaviours and outcomes within P4P schemes.

## Method

For this review, we sought to understand impacts of QOF on the individual clinicians and other groups of professionals in primary care, using a *Lines-of-argument* (LOA) synthesis. The LOA synthesis involves building up a picture of the whole from the studies of its parts [14] and assists knowledge synthesis through a process of re-conceptualisation of themes across several published qualitative papers [14, 15] and is a interpretative approach.

**Fig. 1:**
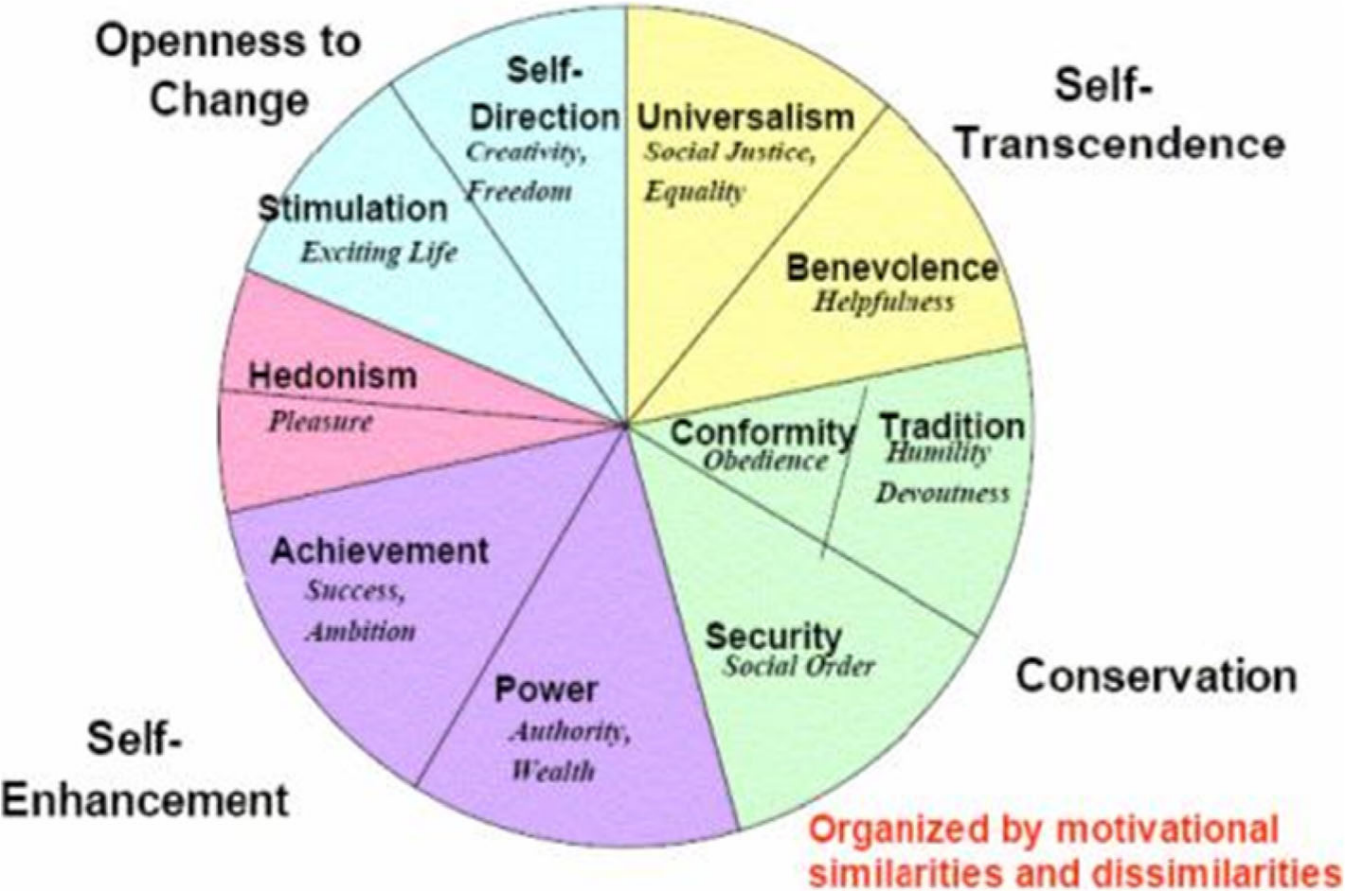
Theoretical Model of Relations Among the Ten Motivational Types of Values [18, 19]**.** Schwartz’s theory identifies ten such motivationally distinct values and further describes the dynamic relations amongst them. In addition to identifying the ten basic values, the theory also explains how these 10 values are interconnected and influence each other, since the pursuit of any of the values results in either an accordance with one another (conformity and security) or a conflict with at least one other value (benevolence and power). (Source: https://www.sciencedirect.com/science/article/pii/S0065260108602816)

We then applied the Schwartz Value Theory as a theoretical framework to our synthesis. Schwartz proposes that there are ten broad Value Domains that are universal and fairly comprehensive [16]. The theory defines these ten broad Values according to the motivation that underlies each of them (described in Table 1) [17]. Although the theory discriminates ten Values, it postulates that, Values form a continuum of related motivations (the circular structure in Fig. 1 portrays the total pattern of relations of conflict and congruity among Values, the closer the Values are on the circular structure then that indicates that they are more congruent and the further away they are, indicates that they are more conflicting [20]. The theory explains that among some Values there is conflict with one another (e.g., benevolence and power) whereas others are congruent (e.g., conformity and security) [18]. One basis of the Value structure is the fact that actions in pursuit of any Value have consequences that conflict with some Values but are congruent with others. Also actions in pursuit of Values have practical, psychological, and social consequences for professionals [17] and their profession [21].

**Table 1:** Schwartz Value Theory: The Ten Basic Values

**Openness to change** **Self-Direction**: Independent thought and action—choosing, creating, exploring. **Stimulation**: Excitement, novelty and challenge in life. **Hedonism**: Pleasure or sensuous gratification for oneself.	
**Self-enhancement** **Achievement:** Personal success through demonstrating competence according to social standards. **Power:** Social status and prestige, control or dominance over people and resources.	
**Conservation** **Security**: Safety, harmony, and stability of society, of relationships, and of self. **Conformity**: Restraint of actions, inclinations, and impulses likely to upset or harm others and violate social expectations or norms. **Tradition**: Respect, commitment, and acceptance of the customs and ideas that one’s culture or religion provides.	
**Self-transcendence** **Benevolence**: Preserving and enhancing the welfare of those with whom one is in frequent personal contact (the ‘in-group’). **Universalism**: Understanding, appreciation, tolerance, and protection for the welfare of all people and for nature.

Professionalism is fundamental to good medical practice and so Professor Dame Judy Dacre also states Medical Professionalism has changed and must keep up to date with the demands of modern day clinical practice [22]. It has been postulated that the professional organisation of medical work no longer reflects the changing health needs caused by the growing number of complex and chronically ill patients [21]. The Royal College of Physicians (RCP) redefined Professionalism in 2018, advising its benefits for patients, that it increases the job satisfaction of doctors, makes for superior organisations, and improves the productivity of health systems. The RCP defined Professionalism as ‘a set of values, behaviours and relationships that underpin the trust the public has in doctors’ [22]. They described seven professional roles; doctor as healer, patient partner, team worker, manager and leader, advocate, learner and teacher and as an innovator (Table. 3). The importance of Medical Professionalism has been well documented in the literature [31], together with its effects on the doctors’ relationships with their patients, quality of care, and ultimately health and illness outcomes [32]. For that reason, we further include Professionalism as a conceptual lens to contextualise our analysis in this review [33].

### Search strategy and data extraction

To identify relevant studies, we searched for peer-reviewed empirical research on QOF using the electronic database searching, hand-searching and web-based searching. The following databases were initially searched: Medline, Embase, Healthstar, CINAHL, and Web of Science. We also searched the reference lists of obtained papers. The details of our electronic search are included in the Additional file 1.

We included studies that reported primary qualitative research (in-depth interviews, focus groups, ethnography, observation, reflective diaries, case-studies and reviews containing qualitative analysis) of the QOF published in English between 2004 (when QOF was introduced) and 2018. We excluded studies that did not specifically focus on the QOF, UK and did not involve primary qualitative research methods.

**Fig. 2:**
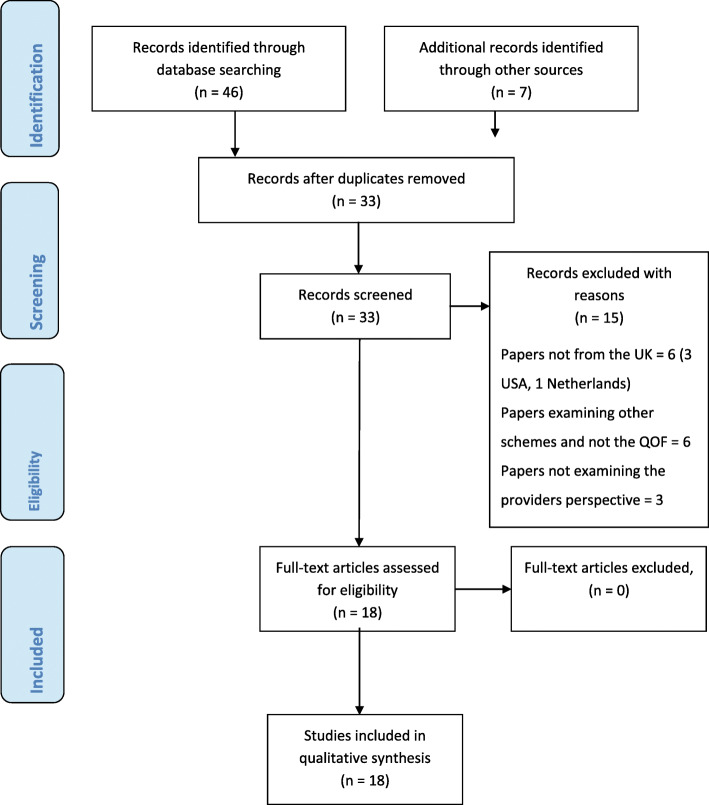
PRISMA Flow Diagram

The search of electronic databases identified 33 relevant papers (see Fig. 2, PRISMA flowchart, including reasons for exclusion). We excluded 15 papers and the 18 papers included were independently reviewed by two researchers (N.K and D.R) and any disagreements discussed. We erred on the side of caution and endeavoured to keep all the 18 studies in until the researcher (N.K.) had independently extracted data from these papers and applied the exclusion criteria.

The researcher (N.K.) extracted data and assessed the eligibility criteria for all retrieved papers, which were then appraised by a second researcher (D.R.). Disagreements between researchers’ were resolved through discussion with S.P. Differences between researchers tended to arise because of different understandings of some of the study questions and because of different interpretations of what authors of the papers had written and generally related to the qualitative research methods used. The qualitative papers were initially quality assessed by N.K. using the British Sociological Association for the evaluation of qualitative research papers [34] and if any discrepancies arose then they were discussed with S.P. The scale comprises 20 questions about the relevance of the study question, appropriateness of qualitative method, transparency of procedures, and ethics. In order to make judgements about the quality of papers, we dichotomised each question to yes or no, in a separate table. All the qualitative papers included in this synthesis were published in peer reviewed journals and adhered to transparency of high quality work.

Following the systematic steps of the meta-ethnography approach, we included 18 qualitative research studies for the final qualitative synthesis.

### Data analysis and interpretation

Meta-ethnography is a systematic but interpretative approach to analysis that begins with noting verbatim and coded text in terms of first-order and second-order constructs. Then translation of these constructs were synthesised across papers to form third-order constructs, and finally constructing the synthesis using either reciprocal, refutational, or line of argument approaches [15, 35]. Our data analysis was undertaken using a ‘line of argument’ synthesis which serves to reveal what is hidden in individual studies and to discover a ‘whole’ among a set of parts [15]. This method has previously been adapted for utility in the syntheses of qualitative data in healthcare research [35, 36]. We placed the 18 papers identified in a table that included relevant details of the study setting and research design (see Additional file 2 Table. 4).

**Fig. 3:**
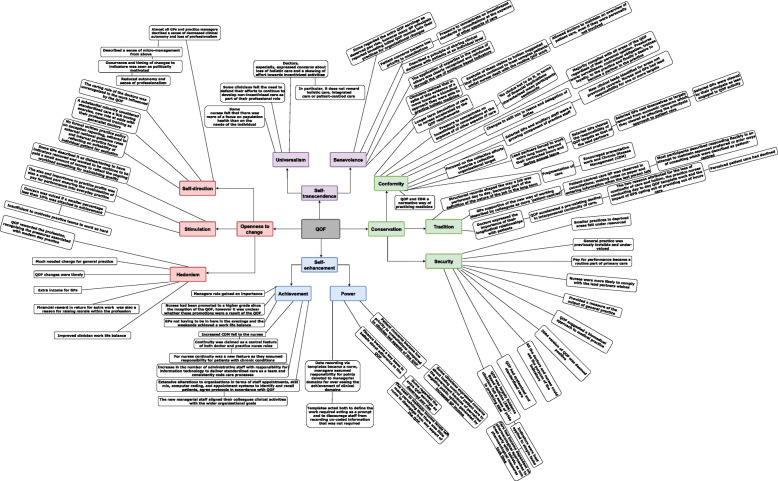
Concepts Mapped to the Ten Values

Our first-order constructs represented the primary data reported in each paper (see Additional File 3 [A3], Table. 5). The emergent themes from the papers represented our second-order constructs (A3, Table. 5). They were extracted utilising a more fine-grained approach, in which the researcher (N.K.) went through each paper in a detailed and line-by-line manner and the papers were reviewed for common and recurring concepts. As a way of remaining faithful to the meanings and concepts of each study; we preserved the terminology used in the original papers in the grids. We then combined and synthesised these themes (taken from the published papers) to create our third-order constructs (see Additional file 4, Table. 6). Each cell of the table was considered in turn, from this, we identified our key concepts and consequent themes and once these where identified (see in an Additional file 5, Table. 7), we simultaneously mapped the concepts against the ten Values using the ten Values as our theoretical framework (Fig. 3). These were then compared with Professionalism as defined by the seven professional roles in Table. 3.

## Results

### Main findings from the synthesis

The 18 papers were published between 2008 and 2018 in the UK. The 18 papers included where of the providers perspective; general practitioners (GP) including GP leads, principals, partners and salaried [23–29, 37–42], nurses [25, 26, 37, 38, 43, 44] (practice and condition specialist) [28–30, 37, 40–42, 44, 45], healthcare assistants [25, 37, 45] and administrative staff [25, 26, 30, 37, 38, 41, 45] (practice managers, IT) on their views and experiences of the QOF. The majority of papers utilised one to one retrospective semi-structured interviews [23, 24, 26, 27, 30, 40–44, 46, 47], focus groups [6, 28, 37, 45, 48], observations [25, 39], using thematic analysis [27, 37, 38, 41], framework approach [26, 44], constant comparison [29, 43] (additionally, see supplementary material 2 for the summary of the sample size, research questions and individual participant characteristics, including the findings from the studies).

The synthesis identified four themes (Table. 2): 1) Loss of autonomy, control and ownership; 2) Incentivised conformity; 3) Continuity of care, holism and the caring role of practitioners in primary care; and 4) Structural and organisational changes. In the next section we present the thematic analysis (summarised in Table. 3) which includes the application of the synthesis to the ten Values with implications for aspects of Professionalism.

**Table 2:** The Impact of the QOF Mapped to the Ten Motivational Values

QOF modifications	Synthesis of the main findings	Influence on ten basic values [18]
Templates Guidelines Indicators Governmental goals	***(a) Loss of autonomy, control and ownership*** Most papers described a sense of decreased clinical autonomy and loss of professionalism [39]. They also described a sense of micromanagement from above [28] and frequently cited the late communication about changes to the wider QOF and year-on-year variability in the occurrence and timing of changes to indicators as politically motivated [28, 39].	***Congruent*** Power Conformity Security Achievement, *Conflict* Self-direction Stimulation Benevolence, Universalism Hedonism, Tradition
Raised standards in basic care Drove provider care Systemized and standardised care Neglected areas of care targeted	***(b) Incentivised conformity*** In the papers reviewed professionals recognized that QOF had led to considerable extra income at the practice level [29]. As the owners of their organizations, economic factors were more salient and apparent in principals’ accounts. Subsequently the finance and achieving maximum income became an increasingly key issue in participants’ beliefs about QOF and their adherence to QOF work [28].	***Congruent*** Achievement Conformity Security Power Tradition *Conflict* Self-direction Stimulation Benevolence Hedonism Universalism
Focus on chronic disease management Certain aspects of professionalism threatened Indicators conflict -patient advocate	***(c) Continuity of care, holism and the caring role of clinicians in primary care*** Although participants in the papers reviewed emphasised the importance of traditional general practice values, such as holism and continuity, the majority felt that the 2004 changes had negatively impacted on these values. Participants related that patients now experienced less continuity with their GPs [41].	***Congruent*** Conformity Power Security Achievement power ***Conflict*** Benevolence Universalism Self-direction Stimulation Tradition
Information technology (IT) Practice managers Increased skill mix Monitoring systems Recording performance Surveillance	***(d) Structural & organisational changes*** All the practices that were studied in the papers included in the review had changed their modes of operation in response to the QOF [27, 29, 43, 45]. Role of monitoring compliance with the coding regime which feeds into the contract monitoring system and of highlighting deficient coding and recording performance amongst staff, contributed to on-one-hand to increased surveillance and on the other to the doctors sense of self-worth [45].	***Congruent*** Power Conformity Achievement Security Stimulation Self-direction Universalism ***Conflict*** Tradition Benevolence Hedonism

**Table 3:** Application of the Synthesis to the Values and Implication for Professionalism

Main Themes	Application of the findings to the Values	Implications for aspects of professionalism [22]
***(a) Loss of autonomy***	***Activated values*** When values are stimulated, they become infused with feeling. Therefore, GPs for whom independence is an important value may experience provocation if their independence (self-direction) seemed to be threatened, discouraged when they are helpless to keep their professional autonomy (power), and would be happy when they can enjoy their freedom as self-regulated practitioners (security).	***Doctor as manager and leader*** Loss of autonomy impacts clinical engagement and leadership which is pivotal to the success of health systems. Doctors make decisions that determine where resources flow. Yet there is a conflict experienced between doctors as employees of huge complex systems and the autonomy of individual doctors. Autonomy is crucial for the delivery of care, but modern autonomy is more complex and nuanced and needs greater judgement [22].
***Control and ownership***	Professionals appeared preoccupied by their lack of control in achieving indicator targets (achievement), especially if dependent upon patient cooperation, quality of care (security), and implementation of outsider perceived changes (power) [23, 24].	***Doctor as team worker*** Relinquishing control is important to allow an important component of teamwork as professional satisfaction, engagement, and effective teamwork improves patient outcomes and satisfaction, as well as organisational performance and productivity. Teamwork has become more important because of the growing complexity of patients’ problems and health systems, and the increasing range of possible interventions [22].
***(b) Incentivised conformity***	***Motivating actions*** Those GPs for whom social order, justice, and medical superiority (power, achievement, and security) are important values are motivated to pursue these incentivised goals (self-satisfaction) in the context of pay for performance schemes. GPs’ values form an ordered system of priorities that characterise them as individuals and general practitioners (professionalism) with specialist set of values, behaviours and relationships that underpin the trust the public has in doctors [22] (tradition, benevolence, universalism, tradition). GPs that hold expert positions as generalist medical practitioners are seen as first point of contact for patients in healthcare services (power, security). They offer a doctor patient relationship with mutual understanding of problems that are brought into the practice (tradition, benevolence, universalism).	***Doctor as advocate*** Professionalism requires that doctors’ advocate on behalf of their patients, all patients and future patients, yet incentivised conformity and indicators conflicted with this aspect. However, this was one concern that should be given the highest priority to advocate on patient safety. Raising concerns about poor care, or the potential for poor care, is a professional duty for all doctors but is not easy; such advocacy needs training, practice, and mentorship [22].
***(c) Continuity of care, holism and the caring role of clinicians in primary care***	***Consequences of cherished values*** Holism and continuity of care (benevolence) for example are relevant in the workplace for GPs (universalism). There was a tension between the standardised QOF driven care, being ‘patient-centred’ with clinicians reporting that “it’s not always easy to deal with disregarding, or setting aside a patient’s’ perceived need or to move onto a more pressing practice target (conformity) during personal discussions” [23, 25–30]. The trade-off between relevant, competing values guides attitudes and behaviours. When values are shown to be in conflict, not corresponding to the cherished value, then do practitioners attribute more importance to their achievement (completing QOF targets, case finding etc.) or justice (work in best interest of others, benevolence, universalism), and to novelty or tradition (medical model). Any attitude or behaviour typically has implications for more than one value. For example, A ‘tick box’ approach to medicine encouraged by pay for performance indicators might express and promote EBM and conformity values at the expense of hedonism and stimulation values for GPs. Values influence action when they are relevant in the context (specific) – such as in pay for performance (hence likely to be activated) and important to the GPs (Status, professional progression, and EBM – achievement, power, security) and bureaucrats (focus on GPs performance to the QOF targets-conformity).	***Doctor as patient partner*** The patient–doctor relationship is at the core of the doctor’s work. The traditional relationship of patient deference to doctors has been replaced by an equal partnership. Values, including integrity, respect, and compassion must underpin the partnership with patients. Integrity involves staying up to date, but also being willing to admit one’s limitations. Doctors can show respect for patients by listening to them actively, involving them in decisions, and respecting their choices (patient centred). Compassion means not just recognising the suffering of the patient, acting to reduce the suffering [22].
***(d) Structural & organisational changes***	***Multiple values*** Values guide the selection or evaluation of actions, policies, people, and events in practice organisations. Hence, GPs in self-regulated disciplines (self-direction) decide what is good or bad, justified or illegitimate, worth doing or avoiding, based on possible consequences for their cherished values. But the impact of values in everyday decisions is rarely conscious and activates a multiple set of values. The results show GP values entered awareness when the QOF actions or judgments GPs were considering had antagonistic or conflicting implications for multiple values they also cherished. Such as undertaking templates use (IT) during consultations. GPs are guided by professional practice which is regulated by the guidelines agreed by GPs. They work to a degree, autonomously although subject to audit and some monitoring. QOF impinges by directing activity in a standardised way (conformity, power).	***Doctor as innovator*** The challenge for doctors is how to innovate amid the innovation happening all around them. The use of machine (in this context -template) learning was feared could lead to the diluted face-to-face patient doctor consultations with a collaboration in which the machine (template) becomes effectively an independent actor. It is doctors, rather than machines, who can provide solidarity, understanding, and compassion to patients [22].

### Loss of autonomy, control and ownership

We found that this theme identified from the published papers [6, 25–28, 30, 42] included professionals’ submission to the QOF targets despite their applied concerns. Such as the ethical distress caused by a reductionist approach to managing markers of chronic disease and its being incompatible with the humanitarian values of general practice [49]. For instance most health professionals believed that they needed to place biomedical care in the context of their patients’ concerns and life experience [50]. We also found that professionals wanted to retain control and clinical autonomy; however on closer examination and within the context of the QOF this took the form of modifying the way structured tools of the QOF were utilised by the professionals.

“The more templates that get introduced, it takes away the clinicians freedom and that sort of rapport that you can build with a patient is much more difficult when you have to go through set (depression score) questions.” (p. 413 ) [42]

“…but I don’t particularly like them... because I tend to write my notes and then do everything on the computer when the patients gone.” (p. 57) [45]

Both, professionals and patients were aware of the QOF targets acting as an independent mechanism of control, which essentially changed the nature of the discussion between patient and professionals [26, 29, 44, 45].

“Some patients will come to you and they’ll plead with you, ‘Please don’t give me any tablets, I’ll bring my blood pressure down, I’ll do anything. I’ll bring it down’, and again they’re not horrendously high, they’re like say 140/90 or whatever … but we’re saying to them ‘well, look we’ve checked it three times now and it remains raised, you’re clinically classed as hypertensive, we follow these guidelines and this is what we should be doing with you.” (p. 143) [25]

Nearly all the published papers showed that the main motivation for practice staff to follow the QOF targets was the link with income loss [23, 24, 26, 29, 40–45].

“So if you deviate from that [QOF] because of the individual need. You have complete autonomy to, but there are financial implications to you because of that…So you still have autonomy, but you lose income.” (p. 57) [24]

This created a conflict for practice staff and suggested a decreasing sense of clinical autonomy. Especially in areas that were clinical and easy to measure and were bound by templates or driven through the use of IT tools [24, 42]. Respondents in one of the papers suggested that most of the internationally agreed attributes of medical professionalism were not perceived or described as being threatened by the introduction of the QOF [42]. Although, on further analysis we found that acquiring a say in the development of indicators was important to GPs and was linked to freedom to practise in the patient’s best interest, indicating that aspects of Professionalism were being affected [22].

### Incentivised conformity

The papers indicated that extensive improvement in QOF scores was perceived as a result of consistency and recording of incentivised activities, the outcomes and new protocols being introduced within practices and that these were now connected to the wider governmental objectives through the mechanism of the QOF [23, 24, 26, 29, 40–45].

“…There are lots of systems in operation here that other people are operating.” (p. 53) [45]

“It’s raised standards, narrowed health inequalities, and introduced evidence-based medicine and err the rest of the world look up on err us and our implementation of QOF with a degree of envy. Its evidence-based medicine, standardised care.” (p. 412) [42]

Respondents in papers reviewed, also stated that the incentive payments attached to QOF did drive provider behaviour and that it encouraged them to work towards performance targets [23–26, 29, 40–45].

“They’re trying to control our income and we’re trying to get as much money out of them as we can.” (p. 412) [42]

Financial rewards in return for extra work was felt to have increased morale for some within the profession [23, 25, 30, 40].

“We’re so hard up at the moment, so desperate for income wherever we can get it, you can’t afford to pass up a chance of income, so that’s probably as much a driver . . . even if we didn’t necessarily buy into the clinical benefit, it was worth doing to try and earn the money because we needed to.” (p. 7) [26]

**Fig. 4:**
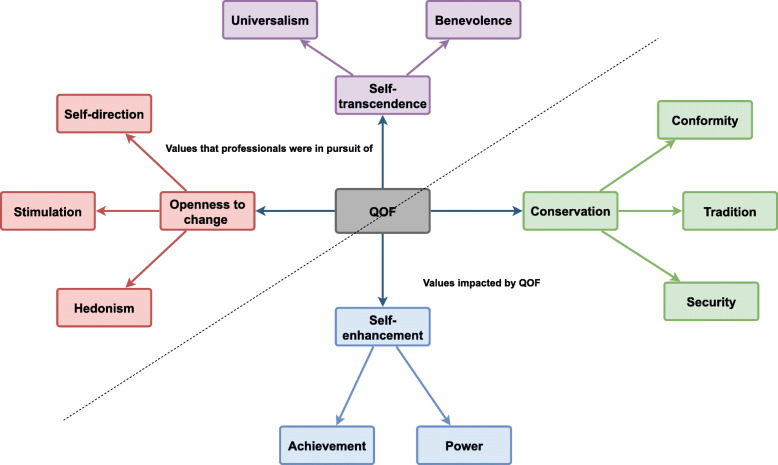
The values Impacted by the QOF

**Fig. 5:**
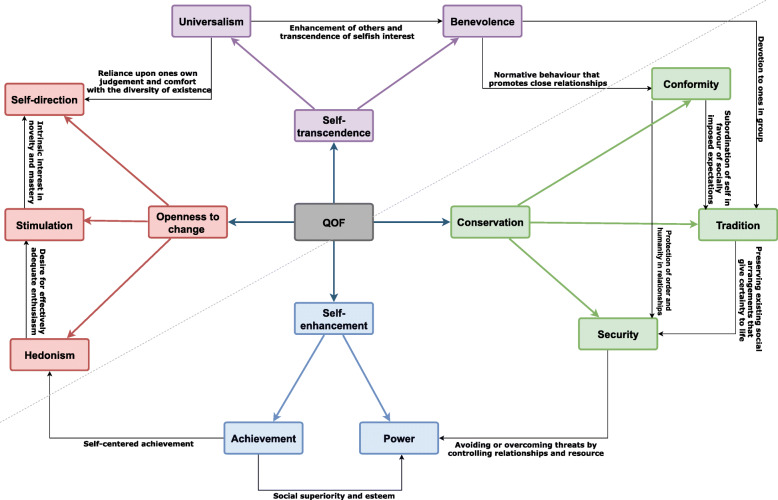
Values that were Congruent with the QOF and Values that were Aligned with Professionalism

Practices had experienced rising practice income and our synthesis findings indicated that certain Values were enhanced by this, particularly power, achievement, conformity, security, and tradition values (Fig. 4). Future P4P schemes should aim to support Values such as benevolence, self-direction, stimulation, hedonism and universalism, which professionals ranked highly and have shown to have positive implications for Professionalism and the efficiency of health systems (Fig. 5). Correspondingly, lower job satisfaction was associated with intention to leave general practice [51]. The papers in the synthesis suggest that the rising income was also linked to the practices adherence to the QOF as a factor that led to the gradual routinisation of the scheme into everyday practice increasing systematised and standardised care [25, 26, 29, 30]. It was also acknowledged that some aspects of neglected clinical activity were appropriately targeted by QOF.

“Patient care has definitely improved because we’ve been doing that, and so I think some people believe we’re number crunching but I don’t think we are in this practice, I think we are actually meeting targets the patients’ care is benefiting.” (p. 52) [45]

Therefore, any changes to QOF are and will be controversial mainly because they represent a substantial proportion of general practitioners’ incomes [52]. Setting the political machinations to one side (the Department of Health has been clawing back from the original settlement since 2004); Gillam and Steel believe that the incentive payments in the QOF also comprise too large a proportion of general practice income. They suggest that money should be taken out of the QOF and redirected to supporting general practice in other ways [52]. However, there is no link between the size of the financial incentive and likely health gain from the activity incentivised [53].

There was also a greater acceptance of standardised approaches [23, 25, 29, 30, 40] which may have restricted personalised care for the individual patient. Even complicated the management of multiple conditions over time [52] and narrowed the focus of the consultation, reducing the time to deal with the wider context of the illness [37]. Further confounded by very limited access to specialist input for patients with more complex treatment resistant or recurrent mental health problems [37].

“We developed this zero tolerance to blood pressure a while ago, no one is allowed to say it’s a little bit up leave it, it’s not acceptable so it has to be if it’s up do something about it, if you’re not doing something about it because if we go and find they’re not on target and you look and they’ve seen somebody and they’ve not acted on it yeh, I’ll have a little word.” (p. 55) [45]

“…the interesting thing for me is that since the introduction of PHQ-9 I find in terms of material I’m treating the score, not the patient. Because, you know, it’s such a short barrier in the consultation.” (p. 282) [37]

Yet, this does not diminish the ethical imperative to practise in the light of best evidence and the challenge is to deliver good quality technical care for medical conditions while simultaneously considering what is in the best interests of the whole person [52].

Some of the QOF’s design flaws are inherent to all pay for performance schemes [54]. As such, areas of high performance will continue to elicit negative feelings, arising from scepticism about achieving maximum points [23, 25, 29, 30, 40].

“I think it’s anyone who gets maximum points is probably bent, I think it’s almost impossible to get maximum points without some kind of fudge. That maybe unkind but we haven’t got maximum points. . . I think its easy just to tick the boxes when you haven’t done it.” (p. 137) [44]

Time pressures were reported to be the motivating factor for prioritising areas of care that were financially incentivised [30].

“I think because there is limited time and if you have to focus on something in order to get the money, obviously if you don’t have the time, then it’s going to be ignored automatically.” (p. 1059) [30]

### Continuity of care, holism and the caring role of practitioners in primary care

Continuity of care, was a central feature of both doctor and practice nurse roles. Organisational and structural changes were attributed to the loss of continuity of care; consequently, accessing the same GP was difficult for patients.

“Increased staff numbers and changed working patterns had contributed to a loss in continuity of care and choice of who to see. The appointment targets paradoxically seemed to have made access worse in many practices, due to requirements to book on the same day . . . We’ve had to have increased staff and then you very quickly lose continuity if you’ve got a lot of people waiting.” (p. 136) [44]

For most nurses, interpersonal continuity was described as a relatively new feature of their role as they assumed further responsibility for patients with chronic conditions [40].

“…with asthma, the patients are beginning to see the same nurse, you know, rather than a different GP… I will see the diabetics and they know that I’ve been trying to say to them, ‘Can you come, you know you can always come back,’ and I always try and make it so that there is open access for them if they have got a problem.” (p. 230) [40]

Holistic care and the caring role of GP practitioners was not recognised in the QOF despite this being seen as a core component of clinical professional roles [22]. Patient-centredness was deemed to be of pronounced significance in the papers reviewed [28, 29, 39, 43]. However, there was a tension between the standardised QOF driven care and being ‘patient-centred’ with practitioners reporting that ‘it’s not always easy to deal with disregarding, or setting aside a patient’s’ perceived need or to move onto a more pressing practice target during these personal discussions’ [24–26, 28–30, 40].

“I tend to deal with the problem patients come with first. And then if it’s appropriate to ask questions, you know, ticking the boxes, I will do that at the end of the consultation.” (p. 231) [40]

“We spend a lot of time visiting... and yet frequency of home visits doesn’t get QOF points ... Caring, that’s what doctors do.” (p. 136) [43]

Papers showed that GPs were more likely to exception report indicators they perceived as having relatively little systematic evaluation or that they were not proven to work. They felt the indicator was contrary to their role as a patient advocate and in their clinical judgement, not relevant to individual patient-centred care [46]. Patient-centredness was defended by professionals in ‘everyday practice’, given the relevance to patient care and the patient-doctor relationship [52, 55–58].

“…Well I think it has put a lot of strain on the partners and practices to get all the QOF points … I mean when it came to get all these points just to get more money, I think it’s put more strain on doctors and it has lost the … just normal care for patients, taking them as a patient rather than as another … object to get points.” (p. 283) [23]

“I think that the art of the job has declined and, I don’t know, the sense of feeling that you could be with people rather than be doing. It’s quite hard to define but there’s more to general practice than doing ... clinical things.” (p. 136) [43]

The synthesis indicates that the QOF embodied an approach to achieving evidence-based medicine (EBM), yet we found no evidence in the papers that linked the compatibility of EBM with a more holistic approach to patient-centred care, as perceived by the professionals and as linked to achieving aspects of Professionalism.

### Structural and organisational changes

QOF was viewed as increasing the responsibility of lead partners (doctors) in most areas of their practice. This included supervising the work of nursing colleagues, which was seen as an increase in their workloads [26, 28, 40, 44].

“There is an environment and ethos of increased surveillance and performance monitoring.” (p. 232) [40]

“I suppose it feels like I’m being watched. It’s a bit like big brother – you’ve not ticked these boxes.” (p. 232) [40]

For some, this has come at the expense of work life balance which manifested as an astonishment with the way their selected profession grasped such issues [24, 43, 44].

“My practice does not understand the concept [work life balance]. And I, we’ve two or three away days a year, I’m often talking about it. And they don’t understand. They’ll take me aside and ‘what do you mean?’ I just find that astonishing you know…, if you have a bereavement of this or that, you just get on with it basically and you don’t expect to be sick for anything… So I mean its just life I’ve chosen, it’s very busy but I do manage to stay sane through it.” (p. 54) [45]

Salaried GPs carried less responsibility for QOF activity than the QOF leaders in areas such as surveillance of others, meeting targets on time, and for the business side of the practice [23].

“I think the balance of, of that is [partners] have a lot more responsibility...you have to take a lot more responsibility for the practice and more leadership. And I quite enjoy ... coming in doing the job and, and not having to worry about that so much. And you get paid more money but I think the balance of the hours you’d be spending and their stress of the job would probably be higher as a partner.” (p. 284) [23]

Those who eventually wanted to succeed to GP principal status took greater responsibility for QOF activity from those who wanted to remain salaried [24, 40, 45].

“But sometimes you do feel that you are not really involved in decision making. That’s fine for some people, but for me, I do like a bit of control. So I think at the moment its fine, but I think eventually I would want part of the decision making process.” (p. 285) [23]

We found that the QOF also impacted the role of nurses but not entirely in same way as it did their GP Colleagues [43, 44]. Nurses initially perceived the changes to their role to be beneficial, which led to professional progression (related to achievement values), however not to any greater authority or any increase in status, which for their GP colleagues were achieved through alignment with the QOF income.

“I’m not comparing it [GP salary] to what the papers say they were walking off with, but (they got) financial rewards for a lot of the work that has been done by nurses.” (p. 714 ) [43]

P4P schemes have been focused on certain medical professionals that make up the healthcare workforce, and the incentives were focused on rewarding those professionals. Our analysis indicates that the QOF work was distributed throughout primary care practice, involving nurses, managerial staff and healthcare assistants but without monetary reward for these groups [28, 43, 44] and this was experienced by other practice staff as an injustice in the reward system. Yet, the effect of income inequality on population health status continues to be described and the link between population health status and socioeconomic status has long been recognised [57] however this link was discounted by the scheme.

Our analysis also showed that except for certain medical professionals, all other groups that made up the primary care staff adhered to the targets without the incentivised reward. As such monetary gain was not the only powerful determinant of employee motivation or positive returns in terms of the QOF performance and success. We also found that the QOF changes for nurses were experienced in isolation of their self-interest and power values, or formal rank (specific to the Nursing discipline), inferring a feeling of continued inequity in primary care practice and healthcare systems.

“Because the workload had increased particularly monitoring wise. We needed to do an awful lot more monitoring of the routine measures. So the combination of that, plus the fact that our nurse had done the diabetes course and asthma course and a prescribing course, we felt that she could move on to something a bit more senior and someone else [the new healthcare assistant (HCA)] could do the routine blood pressures and bloods.” (p. 56) [24]

As a consequence of achievement and increase in workload, the papers in the synthesis revealed that there was an increased blurring of the boundaries with other medical tasks and between different practitioners [25, 28, 43, 44].

“I do in fact do most of the work for the contract and in many ways that’s not a good thing as it is supposed to be team work.” (p. 714) [43]

“... We do the work, the doctor gets the rewards and it is up to him whether he decides to pass it on or not because he gets the global sum now. So that is a bit of a conflict with a lot of the nurses at the moment. So our role and responsibility has expanded but at the same time the wages are staying much the same.” (p. 714) [43]

IT systems were seen as a beneficial tool to help professionals as a form of a reminder, to manage record and collect relevant patient illness related data. But on the other hand, it was a system that made visible the performance of professional work against what was increasingly experienced as ‘outsider implemented targets’. It was not perceived as well by the professionals [23, 40, 44, 45] as there was little scope for the professionals’ to retain personal beliefs or to include patient agendas during reviews [26, 29, 43, 44].

“The more templates that get introduced, it takes away the clinicians freedom and that sort of rapport that you can build with a patient is much more difficult when you have to go through set [depression scores] questions.” (p. 413) [42]

### Application of the findings to the Schwartz value theory and implications for professionalism

In addition to identifying ten basic Values, the Values Theory explicates a structure of dynamic relations among them (see Fig. 1). One basis of the value structure is the fact that actions in pursuit of any Value have consequences that conflict with some Values but are congruent with others. Essentially, choosing an action alternative that promotes one Value (e.g., following template work—conformity) may literally contravene or violate a competing Value (disregarding a patients concerns—benevolence). When we think of our values, we think of what is important to us, each of us hold numerous values (e.g., achievement, security, benevolence) with varying degrees of importance [18]. Furthermore, those actions in pursuit of some values alone had practical, psychological, and social consequences for professionals.

Participants in some papers stated that most of the internationally agreed attributes of Medical Professionalism were not perceived or described as being threatened by the introduction of pay for performance [42]. Contrariwise, the findings of the synthesis revealed that there was some discord experienced by practitioners with some aspects of Professionalism which we present in this section (See Table. 2).

### Triggered values: relinquishing control and retaining independence

Complexity of both patient problems and health systems now requires professionals to work as an interrelated team within the newer hierarchies and hence a relinquishing of control in achieving QOF targets. Initially the issue of retaining control in making decisions in clinical practice was seen as a contentious issue. The concerns were especially regarding who, which or where the body of evidence that was influencing ‘everyday clinical decisions was originating from’ [24, 45]. Other concerns were about government regulation and its influence on the process of care and protecting the well fare of patients and their treatment [24, 45]. Schwartz argued that when Values are triggered, they become infused with feeling [16]. For instance, Schwartz posited four steps in the activation of personal norms that apply equally to basic values [17]. These steps include, awareness of need, awareness of viable actions, perceiving one-self as able to help and then triggering a sense of responsibility to become involved. The synthesis indicates that the introduction of QOF targets influenced behaviour of professionals and it was the operative feature of the targets that triggered the Value for independence linked to the welfare of patients and the care they received (self-transcendence value). Consequently, it was the tension experienced by GP’s in routine practice between their accountability and role requirements under the QOF conditions, which indicated a decrease and loss of professional autonomy. It is important to acknowledge that professional autonomy is recognised by the Royal College of Physicians as a core professional value (Table. 2) [22]. Our analysis proposes that GPs further experienced self-restriction, hierarchical struggle, and outsider control due to the tension imposed by the QOFs influence on the development of indicators. In particular, the Values that were aligned to Professionalism, such as self-direction and stimulation seemingly were experienced as opposing the security, conformity and tradition values, supported by the QOF (Fig. 3). As a result, the restrictiveness of the self-direction Value may have led to the triggering of these conflicts. There were other aspects of the indicators where medical professionals themselves had limited influence (e.g. patient co-operation and access), which further challenged their confidence in achieving the QOF targets [26, 42, 46] causing concern.

### Incentivised conformity in driving the required actions

Typically, people adapt their values to their circumstances [59] and they successively upgrade the importance they attribute to values they can readily attain and downgrade the importance of values whose pursuit is blocked [59]. When the QOF was first announced, primary care had been underfunded, there were large variations in quality between doctors, and general demoralisation within the primary care workforce [60]. Studies in our review suggest that QOF related behaviours raised the profile of general practice (achievement, power, status). This (already) set context may have also contributed to high QOF opt in rates (voluntary) for this P4P scheme in general practice. However, upgrading attainable values and downgrading thwarted values applies to most, but not to all values [55]. We found that Values that concern material well-being achievement, power and security were particularly aligned to the QOF. We also found evidence that when such Values were obstructed, their importance increased and when they were easily attained their importance dropped [61].

“Well it’s certainly improved my income. Probably increased my workload, not to the same degree as it increased my income. But I’m a bit worried that we’ve sold our soul to the devil to some degree, because they can change the goal posts later.” (p. 230) [40]

The presence of the QOF was a requisite and binding, so despite having the choice to opt in, ‘no way out’ of QOF was experienced by those that were in specific QOF leadership roles [24]. Those GPs, for whom social order, justice, and helpfulness in the specific context of the QOF work were important values, would ideally be the target individuals and therefore most likely be motivated to pursue the incentivised goals in the context of this P4P scheme. This however, was experienced by others as confusing in relation to their role of the professional as a patient advocate. For example following a form of prescriptive QOF work was experienced as, taking away time to listen to patient concerns [29] which were perceived as participating in a form of ‘poor’ or ‘low value’ patient care impacting the patient-doctor relationship. RCP suggest this aspect of Professionalism requires training, practice, and mentorship to highlight such antagonisms in patient care [22]. Marcotte et al., propose physicians can and should embrace professionalism as the motivation for redesigning care. Payment reform incentives that align with their professional values should follow and encourage these efforts; that is, payment reform should not be the impetus for redesigning care [62].

### Significance of cherished values; continuity of care, holism and the caring role of clinicians in primary care

Values guide the selection or evaluation of actions, policies, people, and events. Therefore, medical professionals work in self-regulated disciplines, where the profession sets out the parameters of what is good or bad, justified or illegitimate, worth doing or avoiding, based on possible consequences for their cherished values [22] that are related to their profession. However, the impact of Values in everyday decisions is rarely conscious, power values can conflict with universalism and benevolence and these were evident in the accounts of professionals’ which resulted in high arousal to maintain professional behaviours that were linked to their role as patient partners and that were aligned especially to Professionalism (Fig. 5).

“It distracts from the consultations and it can leave you know feeling a bit confused and perhaps as though that, the thing the patients regard as the problem hasn’t been addressed properly.” (p. 8) [26]

The conflicts in Values or changes that were occurring would not have been at the forefront of every professional’s awareness, not until they had started to operate under the QOF conditions or for example when they experienced or became aware of a discontinuity of care for the patients in their daily practice.

“In a sense that it’s still a patient presenting to a doctor with a problem, yes it is the same as it always was. The difference is that it’s more likely that the patient and the doctor won’t know each other.” (p.230) [40]

This highlights the importance of intrinsic motivations [6, 23] for professionals in their day to day work, which if thwarted leads to deepening any individually held disappointment with their profession (satisfaction, stimulation). Recent, GP career intention data has shown that morale had reduced over the past 2 years and intention to leave/retire in the next 2 years increased from 13% in the 2014 survey to 18% 2017 [51]. As a result the theme of personal congruence carried the message that the internal values of a doctor should match the external behaviour and actions [63].

We found that the QOF work was more amenable to the values under conservation and self-enhancement dimensions, and hence directly opposed to the values under self-transcendence and openness to change dimensions (see Fig. 4). As a result, practitioners who were self-directed and worked for the welfare of patients were constrained in their ability to use knowledge attained from previous interactions (patient agendas) with patients in guiding future consultations. This may have led professionals to view standardised care as a ‘box-ticking’ exercise, and at odds with their professional training and their caring role [30]. Holism and patient-centred care were significant values that were particularly vulnerable to QOF changes.

“I thought that you were supposed to tailor this care to every individual patient and meet patient needs...I think it takes away patient, you know, centred care really...I don’t think people appreciate being phoned up all the time and reminding them to come in and things...rightly or wrongly [lead partner] strives for perfection and I think sometimes you have to acknowledge you don’t get perfection all the time and whenever you’re dealing with patients and people you’ll never get perfection anyway.” (p. 56) [45]

Some of the papers, described the need of professionals to defend efforts to continue to deliver non-incentivised care as part of their professional role [25, 44, 45].

Initially, some GPs were apprehensive about the consequences of implementation of indicators in ‘everyday clinical practice’ [26, 29, 30]. Furthermore, there seemed to be insufficient governmental, organisational, administrative, executive, and managerial recognition of the link between the ‘doctors on the ground floor’ working in ‘everyday clinical practice’, and the consequences for ‘routine clinical practice’ and for the professional-patient relationships [24, 25, 45]. Acquiring a say in the development of indicators through negotiations between the BMA and the NHS was an important aspect for professionals, linked to freedom to practice ‘in patients’ best interest’ [6, 24, 42, 45].

“I’d like to see performance measures that really reflect the care.” (p. 553) [46]

“...Some things are ...within the control of the providers, but some things really aren’t, even done ... with good intent.” (p. 553) [46]

“...Often what happens with physicians is things are mandated to us and we don’t have any input in...the process of how things some to us.” (p. 553) [46]

Valderas et al., recommends that person-centred care should be a guiding principle for the development of assessment frameworks and quality indicators. As people-centredness is a core value of health systems, which acknowledges that individual service users should be the key stakeholders and their values, goals and priorities should shape care delivery [64].

### Structural & organisational changes: the trade-off between multiple values

The synthesis showed that all practices had changed their styles of operation in response to the QOF [24, 25, 44, 65]. This involved an increase in the number of administrative staff, including those with responsibility for information technology (IT) [25, 65] and the new managerial stratum worked to align clinical activities to the wider organisational goals [24].

The findings from the synthesis also propose that the QOF targets that were aligned to the conservation and self-enhancement values of GPs, had led to extra income and sizable pay differentials at the practice level were the enabling factor that allowed for the vast organisational and structural changes that took place. These changes were described as a success (achievement) for practices and patients.

“…it’s benefitting the patients, that they don’t get missed , they don’t slip through the net, they get their medicines reviewed, they get their blood tests, they’re kept on optimum treatment.” (p. 135) [44]

Subsequently, the threat to status through competition (stimulation) was seen as a motivator [26].

“It does feel a bit like competition with other surgeries, I don’t know how others feel but I wouldn’t like to come last in our locality.” (p. 7) [26]

Yet, those professionals who were motivated to remain self-directed and aligned their behaviours and attitudes to the welfare of patients, experienced restriction in their ability to use the knowledge attained from patient interactions to guide their future consultations.

“So it’s made the two agendas a little bit clearer and I guess you’ve always had a health agenda and mine is probably never been the same, but now that mine is encapsulated by QOF…it’s a bit more blatantly not the same. So I think there is an intrusion there and it’s not an entirely patient-led agenda, because you’ve got things that you want to do that you think are more important.” (p. 231) [40]

Professionals were making the trade-offs among relevant opposing values based on the QOF targets and that these were guiding the attitudes and behaviours of health care providers in their practice. When Values are in conflict, practitioners will often attribute more importance to the achievement of one set of values at the expense of the others. This hierarchical relationship between values also distinguishes values from norms and attitudes that can be followed unfeelingly. Any attitude or behaviour typically has implications for more than one value, for example, a ‘tick box’ approach to medicine encouraged by P4P indicators promoted EBM and conformity values leading to success, achievement and status at the expense of self-direction, hedonism and stimulation values.

## Discussion

This study involved a meta-synthesis of qualitative studies of provider views of the QOF program. We analysed the literature through the lens of Schwartz’s, Theory of Values as a theoretical framework and to contextualise our analysis we also used Professionalism as a conceptual lens. Using this theoretical framework, we found that QOF related work was experienced by providers as incongruent with their self-direction and benevolence values that are pivotal to professionalism as defined by the Royal College of Physicians [22]. This understanding is likely the result of the QOF being experienced as a mechanism of value activation for only certain values (see Table 2). Values affect behaviour only if they are activated [61]. Activation may or may not entail conscious thought about a value and much information processing occurs outside of awareness [61]. The more accessible a value (the more easily it comes to mind) and the more likely it will be activated and because more important values are more accessible, they relate more to behaviour [18, 66]. For policy and decision makers such insights are valuable in terms of designing P4P schemes. In a report on designing incentive payments for quality care the Conference Board of Canada identified three key guiding concepts – getting the right blend of incentives, alignment with health care goals, global experience and human motivation [67]. Also recognising the importance of values the NHS England review of QOF argued that the scheme needed to be to repositioned *“… as a scheme which recognises and supports the professional values of GPs and their teams in the delivery of first contact, comprehensive, coordinated and person-centred care*” [65].

Our analysis suggests that the pursuit of achievement Values in QOF related work was experienced as compatible with the pursuit of wealth, authority, success, and ambition values that were linked to seeking personal success for GPs. This was likely to reinforce and be supported by QOF actions that were aimed at enhancing GPs social position and status. This also included expanding practice activity, size and overall income, which may be considered as organisational success factors by some GPs. Values such as creativity, social justice, equality, benevolence were experienced as restricted as a result of the QOF targets. Accordingly, when Values are activated, they become infused with feeling both positive or negative [18]. Our synthesis has shown that definition of ‘high quality care’ must be accepted by general practitioners for it to be integrated into practice behaviour. If it is merely derived from an ‘outside regulation’ of clinical practice and assembled by an ‘outside agency’ it will not achieve enduring behaviour change [24, 25, 45, 65].

The direct involvement of providers in the definition of ‘high quality care’ could be one mechanism to balance the discord that was experienced with QOF work. Correspondingly, quality improvement initiatives that are constructed and implemented for the patients’ benefit should be compatible with both EBM and encompass a `patient-centred’ approach. Embedding concepts of high quality primary care, such as those highlighted by Mead and Bower which include a biopsychosocial perspective, `patient-as-person’, sharing power and responsibility, therapeutic alliance, and `doctor-as-person’ in quality improvement initiatives may alleviate some of the tensions that have created unease in general practice as a result of the QOF [68]. A recent systematic review has shown that four of Mead and Bower’s dimensions are still relevant today, and ‘coordinated care’ was a new dimension, reflecting increasing complexity of the health care system [69]. This will likely become more significant as integrated care is planned as a more efficient client-oriented health model [70].

The papers in our analysis described the caring role that encompassed softer values such as the pursuit of novelty, change and stimulation values was likely to be seen to undermine the safeguarding of older customs/tradition values of medicine such as the more biomedical care model. Our analysis also demonstrates that the pursuit of traditional values (clinician-centred care, EBM, and templates) is essentially congruent with the pursuit of conformity values as both motivate actions of submission to external expectations (QOF targets). The Values Theory suggests that everyone experiences conflict between pursuing openness to change values or conservation values and between pursuing self-transcendence or self-enhancement values. Conflicts between specific Values (e.g., power vs. universalism, tradition vs. hedonism) are also near-universal [18].

Values serve as standard or criteria and they tend to guide the selection or evaluation of actions, policies, and events. For example, individuals also decide what is good or bad, justified or illegitimate, worth doing or avoiding based on possible consequences for their cherished values [18]. Achieving some kind of balance in this now appears to have been crucial and the evidence suggests that embracing a more complimentary working between the two, with more focus on the combined efforts is more likely to drive successful complex initiatives. Historically, practices have been autonomous in managerial terms and GPs have been traditionally independently minded [71]. They possess a wide range of norms and values, many of which are desirable but some of which may not be suited to the changes required in complex health systems. For this reason, there are obvious tensions within this relationship with regards to the changes that are ‘softly required’ by health system managers [72]. The synthesis suggests allowing practitioners with competing high priority values to be part of quality improvement initiatives, to take on an influencer role within those initiatives, instead of being ‘responsive agents’ [73]. Initiatives need to consider and engage with concerns of professionals as changes occur in health systems, with timely consultation, piloting and prior to implementation [42].

### Strengths and limitations

Meta-ethnography does offer considerable potential for preserving the interpretive properties of primary data [74]. We acknowledge that the qualitative synthesis cannot be reduced to a set of mechanistic tasks, which raises issues of the transparency of the process [75] which we have tried to make transparent. The goal is to increase understanding, leading to greater explanatory effect, rather than to aggregate and merge findings in a kind of averaging process [76]. We did not have the added benefit of access to any raw data (including transcriptions, reflective notes, and author insight about the context of the studies) as some other meta synthesis have done [76]. Yet, Estabrooks and Field (1994) suggest that the recurrence of themes between compared studies adds to validity similarly to triangulation that is another technique, said to ensure soundness in analysis [77]. Pielstick (1998) understands this as using multiple studies and (meta-synthesis does this by definition) [78]. Undertaking a meta-synthesis is a demanding and laborious process, and would benefit from the development of suitable software [73]. However we feel this will help manage the large amounts of data that emerge from the papers but will not add anything to the process of analysis itself.

## Conclusion

The QOF was instrumental in bringing fundamental changes to general practice organisations. Furthermore, these changes have endured and been embedded into general practice institutions, despite ongoing proposed changes to the QOF. As a mechanism for activating and triggering a select set of Values, the QOF is compatible with the pursuit of wealth, authority, success, ambition and achievement that have implications for Professionalism. In its implementation, QOF also created a ‘standardised success model’ for GPs to motivate and implement ‘actions of submission’ to achieve QOF targets. While QOF was aligned with traditional medical values, influenced by clinician centred care, EBM, and clinical guidelines; our analysis suggests that despite conforming to core medical values there were still some dilemmas regarding whether to pursue income and organisational goals above patient-centred practice.

This analysis of the impact of QOF suggests that in order for quality improvement initiatives, such as P4P schemes to be endurable; they need to be compatible with provider values. P4P schemes need to be designed in order to integrate the personal and professional values that professionals’ find are essential to their practice. Professionals’ have shown that they are driven by their views, beliefs, and experiences, and not just by hierarchy and externally imposed constructs. Our review indicates that policy makers and health service planners need to carefully construct schemes in order to prioritise intrinsic professional values rather than rely on extrinsic motivators that show more limited alignment with Professionalism and its professional core values. Research on QOF has identified that use of performance targets has a limited impact on the quality of care and caused some internal conflicts during the process of carrying out the QOF work. In the UK the shift towards quality improvement approaches that are framed by national priorities and allow for professionals to design their improvement approach, may provide a way of harnessing values of professional autonomy and control as well as building on the motivation to develop patient-centred care. Moves to more network (groups of practices) based schemes may require further thinking as they will be a more complex context with potentially differing, and possibly competing, motivations between practices and practitioners. Our review of the QOF recommends valuable insights that provide those designing P4P systems. It also identifies the need for more qualitative research on the implementation of P4P schemes to fully understand their individual and organisational impact. Further research is also needed to more fully understand how schemes can influence practitioners and support high quality care. In particular it is clear that context; in terms the of the wider organisational structure, payment systems and health system design, need to be more fully considered to fully understand the link between financial incentives, behaviour – both individual and organisational, and quality of care.

## Supplementary information

**Additional file 1.** Search Strategy

**Additional file 2.** Table 4. Contextual Information for the 18 Published Papers

**Additional file 3.** Table. 5 First and Second Order Constructs from the Published Papers

**Additional file 4.** Table. 6 Identifying Third Order Constructs

**Additional file 5.** Table. 7 Application of the Third Order Constructs to the Ten Motivational Values

## Data Availability

Data was generated from the published papers that were included in this synthesis. The dataset used and/or analysed during the current study are available from the corresponding author on reasonable request. N.K, D.R and S.P had access to all relevant papers included, customised tables, and data necessary for verifying the integrity of the data and the accuracy of the analysis. All authors read and approved the final manuscript.

## Abbreviations

QOF: Quality and Outcomes Framework
P4P: Pay for Performance
LOA: Lines-of-argument synthesis
BMA: British Medical Association
RCP: Royal College of Physicians
NHS: National Health Service
QI: Quality Improvement
UK: United Kingdom
EBM: Evidence Based Medicine
GP: General Practitioner
ICT: Information and Communication Technology
NICE: Institute for Health and Care Excellence.
