# Supplementary material for: A pay for performance scheme in primary care: Meta-synthesis of qualitative studies on the provider experiences of the quality and outcomes framework in the UK
Source: BMC Fam Pract. 2020 Jul 13;21:142. doi: 10.1186/s12875-020-01208-8 (PMC7359468; doi:10.1186/s12875-020-01208-8)
Supplement: Supplementary file 1 — Additional file 1. Search Strategy [file 12875_2020_1208_MOESM1_ESM.docx]

**Additional file 1**

**Search strategy**

| **Date** | **Activity** | **Hours** |
| --- | --- | --- |
| **Sept 7 2018** | **Ran search in Ovid Medline 1946 - September week 1 2018**  **Imported 21 references to EndNote**  **EndNote library hold 21 references**  **Ran search in Embase Classic+Embase 1947 to 2018 September 6 excluded Medline records**  **Imported 2 references to EndNote - no duplicates**  **Endnote library holds 23 references**  **Ran search in Ovid Healthstar 1966 to July 2018**  **Imported 19 references to EndNote - 19 duplicates removed**  **Endnote library holds 23 references**  **Ran search in CINAHL 2004 - present Sept 7, 2018**  **Imported 4 references to EndNote - 0 duplicates found**  **EndNote library holds 27 references** | **2 Hours** |
| **Sept 7 2018** | **Processed 18 article requests and emailed via servulink** | **.5 Hour** |
|  |  |  |

**Database(s): All Ovid Medline 1946-Present
Search Strategy:**

| **#** | **Searches** | **Results** |
| --- | --- | --- |
| **1** | **"OUTCOME ASSESSMENT (HEALTH CARE)"/** | **64902** |
| **2** | **"OUTCOME AND PROCESS ASSESSMENT (HEALTH CARE)"/** | **25287** |
| **3** | **(outcome adj2 assessment).mp. [mp=title, abstract, original title, name of substance word, subject heading word, floating sub-heading word, keyword heading word, protocol supplementary concept word, rare disease supplementary concept word, unique identifier, synonyms]** | **73860** |
| **4** | **(quality adj1 outcome* adj1 framework*).ti,ab. or Quality Indicators, Health Care/ or quality indicators.mp. [mp=title, abstract, original title, name of substance word, subject heading word, floating sub-heading word, keyword heading word, protocol supplementary concept word, rare disease supplementary concept word, unique identifier, synonyms]** | **17415** |
| **5** | **quality of health care.mp. or "Quality of Health Care"/** | **134011** |
| **6** | **Physician Incentive Plans/ or physician incentive.mp.** | **2197** |
| **7** | **REIMBURSEMENT, INCENTIVE/** | **4014** |
| **8** | **(reimbursement adj3 incentive*).mp. [mp=title, abstract, original title, name of substance word, subject heading word, floating sub-heading word, keyword heading word, protocol supplementary concept word, rare disease supplementary concept word, unique identifier, synonyms]** | **4168** |
| **9** | **(pay adj2 performance).mp.** | **1968** |
| **10** | **ENGLAND/ or england.mp.** | **109179** |
| **11** | **scotland.mp. or SCOTLAND/** | **29467** |
| **12** | **WALES/ or wales.mp.** | **34562** |
| **13** | **united kingdom.mp. or United Kingdom/** | **235696** |
| **14** | **1 or 2 or 3** | **98892** |
| **15** | **4 or 5** | **136554** |
| **16** | **6 or 7 or 8 or 9** | **6870** |
| **17** | **10 or 11 or 12 or 13** | **381637** |
| **18** | **14 and 16 and 17** | **81** |
| **19** | **15 and 16 and 17** | **245** |
| **20** | **18 or 19** | **268** |
| **21** | **limit 20 to english language** | **262** |
| **22** | **((("semi-structured" or semistructured or unstructured or informal or "in-depth" or indepth or "face-to-face" or structured or guide) adj3 (interview* or discussion* or questionnaire*)) or (focus group* or qualitative or ethnograph* or fieldwork or "field work" or "key informant" or observation or "reflective diaries")).ti,ab. or interviews as topic/ or focus groups/ or narration/ or qualitative research/** | **586058** |
| **23** | **21 and 22** | **21** |
| **24** | **limit 23 to yr="2004 -Current"** |  |

**Database(s): Embase Classic+Embase 1947 to 2018 September 6
Search Strategy:**

| **#** | **Searches** | **Results** |
| --- | --- | --- |
| **1** | **outcome assessment.mp. or exp outcome assessment/** | **444264** |
| **2** | **(outcome adj2 assessment).mp. [mp=title, abstract, heading word, drug trade name, original title, device manufacturer, drug manufacturer, device trade name, keyword, floating subheading word, candidate term word]** | **441914** |
| **3** | **quality of health care.mp. or exp health care quality/** | **2785582** |
| **4** | **(quality adj1 outcome* adj1 framework).ti,ab.** | **92** |
| **5** | **quality indicators.mp.** | **9061** |
| **6** | **1 or 2 or 3 or 4 or 5** | **2793041** |
| **7** | **physician incentive.mp.** | **103** |
| **8** | **reimbursement.mp. or exp reimbursement/** | **61430** |
| **9** | **(pay adj2 performance).mp. [mp=title, abstract, heading word, drug trade name, original title, device manufacturer, drug manufacturer, device trade name, keyword, floating subheading word, candidate term word]** | **2552** |
| **10** | **(incentiv* adj5 physician*).mp. [mp=title, abstract, heading word, drug trade name, original title, device manufacturer, drug manufacturer, device trade name, keyword, floating subheading word, candidate term word]** | **1280** |
| **11** | **7 or 8 or 9 or 10** | **64068** |
| **12** | **england/ or england.mp. or scotland/ or scotland.mp. or wales/ or wales.mp. [mp=title, abstract, heading word, drug trade name, original title, device manufacturer, drug manufacturer, device trade name, keyword, floating subheading word, candidate term word]** | **128734** |
| **13** | **6 and 11 and 12** | **261** |
| **14** | **(((semi-structure* or semistructure* or unstructed or informal or in-depth or indepth or face-to-face or structured or guide) adj3 interview) or discussion or questionnaire or focus group* or qualitative or ethnograph* or fieldwork or key informant or observation or reflective diaries).mp. [mp=title, abstract, heading word, drug trade name, original title, device manufacturer, drug manufacturer, device trade name, keyword, floating subheading word, candidate term word]** | **1874081** |
| **15** | **focus groups/** | **182730** |
| **16** | **narration/** | **13269** |
| **17** | **qualitative research/** | **55677** |
| **18** | **14 or 15 or 16 or 17** | **2028298** |
| **19** | **13 and 18** | **67** |
| **20** | **limit 19 to (english language and exclude medline journals and yr="2004 -Current")** | **2** |

**Database(s): Ovid Healthstar 1966 to July 2018
Search Strategy:**

| **#** | **Searches** | **Results** |
| --- | --- | --- |
| **1** | **"OUTCOME ASSESSMENT (HEALTH CARE)"/** | **60801** |
| **2** | **"OUTCOME AND PROCESS ASSESSMENT (HEALTH CARE)"/** | **23319** |
| **3** | **(outcome adj2 assessment).mp. [mp=title, original title, abstract, floating sub-heading word, name of substance word, subject heading word]** | **67384** |
| **4** | **(quality adj1 outcome* adj1 framework*).ti,ab. or Quality Indicators, Health Care/ or quality indicators.mp. [mp=title, original title, abstract, floating sub-heading word, name of substance word, subject heading word]** | **15377** |
| **5** | **quality of health care.mp. or "Quality of Health Care"/** | **127740** |
| **6** | **Physician Incentive Plans/ or physician incentive.mp.** | **2088** |
| **7** | **REIMBURSEMENT, INCENTIVE/** | **3704** |
| **8** | **(reimbursement adj3 incentive*).mp. [mp=title, original title, abstract, floating sub-heading word, name of substance word, subject heading word]** | **3830** |
| **9** | **(pay adj2 performance).mp.** | **1561** |
| **10** | **ENGLAND/ or england.mp.** | **87559** |
| **11** | **scotland.mp. or SCOTLAND/** | **23013** |
| **12** | **WALES/ or wales.mp.** | **27861** |
| **13** | **united kingdom.mp. or United Kingdom/** | **203067** |
| **14** | **1 or 2 or 3** | **90479** |
| **15** | **4 or 5** | **129225** |
| **16** | **6 or 7 or 8 or 9** | **6217** |
| **17** | **10 or 11 or 12 or 13** | **318991** |
| **18** | **14 and 16 and 17** | **72** |
| **19** | **15 and 16 and 17** | **216** |
| **20** | **18 or 19** | **235** |
| **21** | **limit 20 to english language** | **231** |
| **22** | **((("semi-structured" or semistructured or unstructured or informal or "in-depth" or indepth or "face-to-face" or structured or guide) adj3 (interview* or discussion* or questionnaire*)) or (focus group* or qualitative or ethnograph* or fieldwork or "field work" or "key informant" or observation or "reflective diaries")).ti,ab. or interviews as topic/ or focus groups/ or narration/ or qualitative research/** | **339266** |
| **23** | **21 and 22** | **19** |
| **24** | **limit 23 to yr="2004 -Current"** | **19** |

| **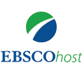** | **CINAHL DATABASE**  **Friday, September 07, 2018 1:30:10 PM** |
| --- | --- |

| **#** | **Query** | **Limiters/Expanders** | **Results** |
| --- | --- | --- | --- |
| **S20** | **S12 AND S15 AND S16 AND S17** | **Limiters - Published Date: 20040101-20161231; English Language; Exclude MEDLINE records  Search modes - Boolean/Phrase** | **4** |
| **S19** | **S12 AND S15 AND S16 AND S17** | **Limiters - Published Date: 20040101-20161231  Search modes - Boolean/Phrase** | **23** |
| **S18** | **S12 AND S15 AND S16 AND S17** | **Search modes - Boolean/Phrase** | **23** |
| **S17** | **S13 OR S14** | **Search modes - Boolean/Phrase** | **576,159** |
| **S16** | **S9 OR S10 OR S11** | **Search modes - Boolean/Phrase** | **2,270** |
| **S15** | **S1 OR S2 OR S3 OR S4 OR S5 OR S6 OR S7 OR S8** | **Search modes - Boolean/Phrase** | **80,393** |
| **S14** | **focus w1 group* OR qualitative OR ethnograph* OR fieldwork OR key w1 informant OR observation OR reflective w1 diaries OR narration** | **Search modes - Boolean/Phrase** | **157,684** |
| **S13** | **semi-structured OR semistructured OR unstructured OR informal OR in-depth OR indepth OR face-to-face OR structured OR guide OR interview OR discussion OR questionnaire** | **Search modes - Boolean/Phrase** | **517,479** |
| **S12** | **england/ OR england OR scotland/ OR scotland OR wales/ OR wales OR united kingdom/ OR united kingdom** | **Search modes - Boolean/Phrase** | **267,139** |
| **S11** | **pay w2 performance** | **Search modes - Boolean/Phrase** | **1,142** |
| **S10** | **reimbursement w3 incentiv*** | **Search modes - Boolean/Phrase** | **1,303** |
| **S9** | **(MH "Reimbursement, Incentive")** | **Search modes - Boolean/Phrase** | **1,254** |
| **S8** | **quality w1 health w1 care** | **Search modes - Boolean/Phrase** | **48,948** |
| **S7** | **(MH "Quality of Health Care")** | **Search modes - Boolean/Phrase** | **47,488** |
| **S6** | **quality w1 indicator*** | **Search modes - Boolean/Phrase** | **2,414** |
| **S5** | **(MH "Clinical Indicators")** | **Search modes - Boolean/Phrase** | **8,098** |
| **S4** | **quality w1 outcome* w1 framework** | **Search modes - Boolean/Phrase** | **224** |
| **S3** | **(MH "Outcome Assessment") OR "outcome assessment"** | **Search modes - Boolean/Phrase** | **25,620** |
| **S2** | **MH outcome assessment** | **Search modes - SmartText Searching** | **460,315** |
| **S1** | **MH outcome assessment (health care)** | **Search modes - Boolean/Phrase** | **0** |

**Web of Science**

| **# 8** | [**3**](http://apps.webofknowledge.com.myaccess.library.utoronto.ca/summary.do?product=WOS&doc=1&qid=8&SID=5FthK2bFDf5S4vjKjSV&search_mode=GeneralSearch&update_back2search_link_param=yes) | **TITLE: (quality outcomes framework)**  **Refined by: PUBLICATION YEARS: ( 2018 OR 2017 OR 2016 OR 2015 OR 2014 ) AND TOPIC: (england) AND TOPIC: (united kingdom)**  **Indexes=SCI-EXPANDED, SSCI, A&HCI, CPCI-S, CPCI-SSH, ESCI Timespan=All years** |  |
| --- | --- | --- | --- |
| **# 7** | [**14**](http://apps.webofknowledge.com.myaccess.library.utoronto.ca/summary.do?product=WOS&doc=1&qid=7&SID=5FthK2bFDf5S4vjKjSV&search_mode=GeneralSearch&update_back2search_link_param=yes) | **TITLE: (quality outcomes framework)**  **Refined by: PUBLICATION YEARS: ( 2018 OR 2017 OR 2016 OR 2015 OR 2014 ) AND TOPIC: (england)**  **Indexes=SCI-EXPANDED, SSCI, A&HCI, CPCI-S, CPCI-SSH, ESCI Timespan=All years** |  |
| **# 6** | [**1**](http://apps.webofknowledge.com.myaccess.library.utoronto.ca/summary.do?product=WOS&doc=1&qid=6&SID=5FthK2bFDf5S4vjKjSV&search_mode=GeneralSearch&update_back2search_link_param=yes) | **TITLE: (quality outcomes framework)**  **Refined by: PUBLICATION YEARS: ( 2018 OR 2017 OR 2016 OR 2015 OR 2014 ) AND TOPIC: (wales)**  **Indexes=SCI-EXPANDED, SSCI, A&HCI, CPCI-S, CPCI-SSH, ESCI Timespan=All years** |  |
| **# 5** | **0** | **TITLE: (quality outcomes framework)**  **Refined by: PUBLICATION YEARS: ( 2018 OR 2017 OR 2016 OR 2015 OR 2014 ) AND TOPIC: (scotland) AND TOPIC: (wales)**  **Indexes=SCI-EXPANDED, SSCI, A&HCI, CPCI-S, CPCI-SSH, ESCI Timespan=All years** |  |
| **# 4** | [**1**](http://apps.webofknowledge.com.myaccess.library.utoronto.ca/summary.do?product=WOS&doc=1&qid=4&SID=5FthK2bFDf5S4vjKjSV&search_mode=GeneralSearch&update_back2search_link_param=yes) | **TITLE: (quality outcomes framework)**  **Refined by: PUBLICATION YEARS: ( 2018 OR 2017 OR 2016 OR 2015 OR 2014 ) AND TOPIC: (scotland)**  **Indexes=SCI-EXPANDED, SSCI, A&HCI, CPCI-S, CPCI-SSH, ESCI Timespan=All years** |  |
| **# 3** | [**46**](http://apps.webofknowledge.com.myaccess.library.utoronto.ca/summary.do?product=WOS&doc=1&qid=3&SID=5FthK2bFDf5S4vjKjSV&search_mode=GeneralSearch&update_back2search_link_param=yes) | **TITLE: (quality outcomes framework)**  **Refined by: PUBLICATION YEARS: ( 2018 OR 2017 OR 2016 OR 2015 OR 2014 )**  **Indexes=SCI-EXPANDED, SSCI, A&HCI, CPCI-S, CPCI-SSH, ESCI Timespan=All years** |  |
| **# 2** | [**153**](http://apps.webofknowledge.com.myaccess.library.utoronto.ca/summary.do?product=WOS&doc=1&qid=2&SID=5FthK2bFDf5S4vjKjSV&search_mode=GeneralSearch&update_back2search_link_param=yes) | **TITLE: (quality outcomes framework)**  **Indexes=SCI-EXPANDED, SSCI, A&HCI, CPCI-S, CPCI-SSH, ESCI Timespan=All years** | [**Edit**](http://apps.webofknowledge.com.myaccess.library.utoronto.ca/WOS_AdvancedSearch_input.do?product=WOS&SID=5FthK2bFDf5S4vjKjSV&search_mode=AdvancedSearch&replaceSetId=2&editState=init) |
| **# 1** | [**9,963**](http://apps.webofknowledge.com.myaccess.library.utoronto.ca/summary.do?product=WOS&doc=1&qid=1&SID=5FthK2bFDf5S4vjKjSV&search_mode=GeneralSearch&update_back2search_link_param=yes) | **TOPIC: (quality outcomes framework)**  **Indexes=SCI-EXPANDED, SSCI, A&HCI, CPCI-S, CPCI-SSH, ESCI Timespan=All years** | [**Edit**](http://apps.webofknowledge.com.myaccess.library.utoronto.ca/WOS_AdvancedSearch_input.do?product=WOS&SID=5FthK2bFDf5S4vjKjSV&search_mode=AdvancedSearch&replaceSetId=1&editState=init) |
