# Supplementary material for: A pay for performance scheme in primary care: Meta-synthesis of qualitative studies on the provider experiences of the quality and outcomes framework in the UK
Source: BMC Fam Pract. 2020 Jul 13;21:142. doi: 10.1186/s12875-020-01208-8 (PMC7359468; doi:10.1186/s12875-020-01208-8)
Supplement: Supplementary file 2 — Additional file 2. Table 4. Contextual Information for the 18 Published Papers [file 12875_2020_1208_MOESM2_ESM.docx]

**Additional file 2**

**Table 4. Contextual Information for the 18 Published Papers**

| **Source** | **Title** | **Aims** | **Method and sample** | **Data collection** | **Participants** | **Findings** |
| --- | --- | --- | --- | --- | --- | --- |
| Mitchell C, Dwyer R, Hagan T and Mathers N (2011) | Impact of the QOF and the NICE guideline in the diagnosis and management of depression: a qualitative study. | To explore primary care practitioner perspectives on the clinical utility of the NICE guideline and the impact of the QOF on diagnosis and management of depression in routine practice. | Qualitative study using focus groups.  Used a topic guide and audiotaping.  Data analysis was iterative and thematic. | 4 focus groups were conducted, (minimum of 8 and maximum of 10 participants.  In 4 multidisciplinary practice teams with diverse populations in south Yorkshire. | 38 participants:  17 GPs,  2 specialist training registrars in general practice,  11 practice nurses,  2 community nurses,  2 doctors in foundation training,  3 primary care mental health workers and  1 manager. | Organisational barriers to the implementation of the NICE guideline and the limited scope of the QOF highlight the need for policymakers to work more effectively with the complex realities of general practice in order to systematically improve the quality and delivery of ’managed’ care for depression. |
| Hannon KL, Lester HE, Campbell SM (2011) | Recording patient preferences for end-of-life care as an incentivized quality indicator: What do general practice staff think? | The aim of this study was to gain the views and experiences of general practice staff on whether the inclusion of a single incentivized indicator to record the preferred place to receive end-of-life care would improve the quality of palliative care. Any issues arising from its implementation in a pay-for-performance scheme were also explored. | Qualitative study using interviews.  Thematic analysis.  Interviewees were recruited from a nationally representative sample of 28 general practices. | Face to face semi structured interviews.  12 Primary Care Trust (PCT) areas in England.  The practices were representative in terms of practice list size, level of deprivation and performance on QOF in the preceding year.  2 practices each from the devolved administrations in Scotland, Wales and Northern Ireland were also recruited. These practices were sampled from a list of interested practices supplied by the NICE. | 57 Interview:  21 GPs,  16 practice managers,  12 nurses and  8 others (mostly information technology experts). | The most appropriate time to ask a patient about end-of-life care is subjective and patient specific and therefore does not lend itself to an inflexible single indicator.  Focusing on one isolated question simplifies and distracts from a multi-faceted and complex issue and may lead to patient harm. |
| Checkland K,  Harrison S (2010) | The impact of the Quality and Outcomes  Framework on practice organisation and  service delivery: summary of evidence from  two qualitative studies. | To investigate in detail the impact of the QOF on practice organisation and service delivery. | Two linked ethnographic case studies of the new contract. Two practices in England and two practices in Scotland. | Interviews and Observation. | Four general medical practices.  Practice one:  4 partners,  3 nurses practitioners,  1 practice nurse,  1 healthcare assistant.  Practice two:  7 partners,  2 salaried GPs,  2 nurse practitioners,  3 chronic disease nurses,  2 healthcare assistants.  Practice three:  3 practice nurses,  1 healthcare assistant.  Practice four:  6 partners,  2 salaried GPs,  3 practice nurses,  2 healthcare assistants. | A number of significant changes to practice organisation and service delivery were observed, including:  Changes to practice organisational structures;  An increased role for information technology;  A move towards a more biomedical form of medical care.  Changes to roles and relationships, including the introduction of internal peer-review and surveillance.  In spite of this, the practices maintained a narrative of ‘no change’, arguing that they had ‘fitted QOF in’ to their routines with little trouble. |
| Hackett J, Glidewell L, West R, Carder P , Doran T, Foy R. (2014) | 'Just another incentive scheme': a qualitative interview study of a local pay-for-performance scheme for primary care. | Investigated whether professionals’ experience of a local scheme in one English National Health Service (NHS) former primary care trust (PCT) differed from that of the national QOF in relation to the goal of reducing inequalities. | Purposively sampled 16 practices with varying levels of population socio-economic deprivation and achievement.  Using a framework approach to analysis. | Retrospective Semi structured face to face interviews were conducted with primary care professionals. | 44 professionals interviewed.  38 practice staff interviewed,  15 practice managers  10 GPs,  2 salaried GPs,  11 practice nurses.  6 – additional participants,  Comprised 4 PCT managers,  1 salaried GP,  1 practice nurse. | The contentious nature of pay-for-performance was not necessarily reduced by local adaptation.  Those developing future schemes should consider differential rewards and supportive resources for practices serving more deprived populations, and employing a wider range of levers to promote professional understanding and ownership of indicators. |
| Alderson SL, Amy M Russell, McLintock K, Potrata B, House A, Foy R. (2014) | Incentivised case finding for depression in patients with chronic heart disease and diabetes in primary care: an ethnographic study. | To examine the process of case finding for depression in people with diabetes and coronary heart disease within the context of a pay-for-performance scheme. | Qualitative study  General practices in Leeds, UK.  Observation field notes were thematically analysed. | Ethnographic study drawing on observations of practice routines and consultations, debriefing interviews with staff and patients and review of patient records. | 12 purposively sampled practices.  119 staff;  63 consultation, observations and  57 patient interviews. | Case finding does not fit naturally within consultations; both professional and patient reactions somewhat subverted the process recommended by national guidance.  Quality improvement strategies will need to take account of our results in two ways.  First, despite their apparent simplicity, the case finding questions are not consultation-friendly and acceptable alternative ways to raise the issue of depression need to be supported.  Second, case finding needs to operate within structured pathways which can be accommodated within available systems and resources. |
| McGregor W, Jabareen H, O’Donnell CA, Mercer SW, Graham, Watt GC (2008) | Impact of the 2004 GMS contract on practice nurses: a qualitative study. | This study investigated how practice nurses perceived the changes in their work since the contract’s inception. | A qualitative study,  Glasgow, UK,  Interviews were audiotaped, transcribed, and analysed using a constant comparative approach.  Sampling practice nurses from practices in areas of high and low deprivation, with a range of QOF scores. | Individual semi structured interviews . | 18 practice nurses. | The new GMS contract has given practice nurses increased responsibility.  However, discontent about how financial gains are distributed and negative impacts on core values may lead to detrimental long term effects on motivation and morale. |
| Gill PJ, Hislop J, Mant D, Harnden A (2012) | General practitioners' views on quality markers for children in UK primary care: a qualitative study. | Children make up about 20% of the UK population and caring for them is an important part of a general practitioner’s (GP’s) workload. However, the UK Quality Outcomes Framework (pay-for-performance system) largely ignores children – less than 3% of the quality markers relate to them. As no previous research has investigated whether GPs would support or oppose the introduction of child-specific quality markers, we sought their views on this important question. | Qualitative interview study  Analysis was thematic and used constant comparative method to look for anticipated and emergent themes as the analysis progressed. | Semi-structured interviews explored GPs’ viewpoints. | 4 primary care trusts in Thames Valley, England.  20 GPs. | The GPs expressed support for the development of quality markers for the care of children in UK general practice.  However, they flagged up a number of important challenges which need to be addressed if markers are to be developed that are measureable, targeted and within the direct control of primary care.  Easy access to primary and secondary care appointments may be an important benchmark for commissioners of care. |
| Cheraghi-Sohi S, McDonald R, Harrison S, Sanders C. (2012) | Experience of contractual change in UK general practice: a qualitative study of salaried GPs. | To explore the views and experiences of salaried GPs working in English general practice. | Qualitative study.  General Practice in England. | Used semi-structured interviews, using a topic guide. | 17 practices across England,  11 Primary Care Trusts,  23 salaried GPs. | Salaried GPs’ working experiences were dependent upon personal aspirations and local context.  Most salaried GPs were reportedly content with their current position but many also had aspirations of eventually attaining GP principal status.  The current lack of available partnerships threatens to undo recent positive workforce progress and may lead to deep dissatisfaction within the profession and a future workforce crisis.  Further large-scale quantitative work is required to assess the satisfaction and future expectations of those in salaried posts. |
| Campbell S, Hannon K, Lester H (2011) | Exception reporting in the Quality and Outcomes Framework: views of practice staff - a qualitative study. | To explore GP and practice staff views and experiences of exception reporting in the QOF. | Qualitative study  Analysed using open explorative thematic coding. | Qualitative semi-structured interviews. | In 27 general practices in the UK.  24 GPs,  20 practice managers,  13 practice nurses,  and  9 other staff were conducted. | Exception reporting is seen by most GPs and practice staff as an important and  Defensible safeguard against inappropriate treatment or over-treatment of patients.  However, a minority of practitioners also saw it as a gaming mechanism. |
| Maxwell M, Harris F, Hibberd C , Donaghy E , Pratt R, Williams C , Morrison J, Gibb J, Watson P, Burton C. (2013) | A qualitative study of primary care professionals' views of case finding for depression in patients with diabetes or coronary heart disease in the UK. | Explored the views and experiences of primary care nurses, doctors and managers to understand how the implementation of case finding/screening might impact on its effectiveness. | Two complementary qualitative focus group studies. Explore similar populations. Combined to achieve a larger sample and a wider range of views. | Focus groups. | 5 primary care practices and  5 Community Health Partnerships were conducted in Scotland.  Primary care professionals.  Study one:  8 practice nurses,  6 GPs.  Study two:  13 practice nurses  14 GPs,  28 Long term condition/specialist nurses. | The introduction of case finding/screening for depression into routine chronic illness management is not straightforward.  Routinized case finding/screening for depression can be implemented in ways that may be counterproductive to engagement (particularly by nurses), with the mental health needs of patients living with long term conditions.  If case finding/screening or engagement with mental health problems is to be promoted, primary care nurses require more training to increase their confidence in raising and dealing with mental health issues and GPs and nurses need to work collectively to develop the relational work required to promote cognitive participation in case finding/screening. |
| Chew-Graham CA, Hunter C, Langer S, Stenhoff A, Drinkwater J,Guthrie EA, Salmon P. (2013) | How QOF is shaping primary care review consultations: a longitudinal qualitative study. | Reports a qualitative study in which the focus is on the ways in which QOF (Quality and Outcomes Framework) shapes routine review consultations, and highlight the tensions exposed between patient-centred consulting and QOF-informed Long Term Care (LTC) management. | A longitudinal qualitative study.  Audio recorded consultations of primary care practitioners with patients with LTC  Analysis of the data sets used a constant comparative approach. | Interviewed both patients and practitioners using tape-assisted recall.  Patient participants were followed for three months during which the research team made weekly contact and invited them to complete weekly logs about their health service use.  A second interview at three months was conducted with patients. | 6 practices agreed to participate,  34 patients recruited to the study,  18 cases with complete set of recordings (consultation, patient baseline interview, patient follow-up interview, healthcare practitioner interview).  16 sets with partial set of recordings.  Total of 88 transcripts were analysed. | Routine review consultations in primary care focus on the biomedical agenda set by QOF where the practitioner is the expert, and the patient agenda unheard.  Review consultations shape patients’ expectations of future care and socialize patients into becoming passive subjects of ‘surveillance’.  Patient needs outside the narrow protocol of the review are made invisible by the process of review except in extreme cases such as anticipating death and bereavement. |
| Lester HE, Hannon KL, Campbell SM (2011) | Identifying unintended consequences of quality indicators: a qualitative study. | This paper describes an in-depth exploration of family physician, nurse and other primary-care practice staff views of the value of piloting with a particular focus on unintended consequences of 13 potential new QOF indicators. | Explorative qualitative  Study.  Used open coding. | Semi structured interviews. | In 24 representative practices across England.  57 family-practice professionals were interviewed.  21 family doctors,  16 practice managers,  12 nurses,  8 other staff IT/computer staff or notes summarisers. | It is important to identify concerns and experiences about unintended consequences of indicators at an early stage when there is time to remove or adapt problem indicators.  Since the UK government currently spends over £1 billion each year on QOF,  The £150 000 spent on each piloting cohort (0.0005% of the total QOF budget) appears to be good value for money. |
| Maisey S, Steel N, Marsh R, Gillam S1, Fleetcroft R, Howe A. (2008) | Effects of payment for performance in primary care: qualitative interview study. | To understand the effects of a large scale ‘payment for performance’ scheme (the Quality and Outcomes Framework [QOF]) on professional roles and the delivery of primary care in the English National Health Service. | Qualitative study  Framework Analysis approach.  Thematic framework. | Semi-structured interviews. | In 12 general practices in eastern England with a broad range of sociodemographic and organizational characteristics.  Carried out interviews with 1 doctor and 1 nurse from each practice.  24 clinicians were interviewed. | Payment for performance is driving major changes in the roles and organization of English primary health care teams.  Non-incentivized activities and patients’ concerns may receive less clinical attention.  Practitioners would benefit from improved dissemination of the evidence justifying the inclusion of new performance indicators in the QOF. |
| Campbell SM, McDonald R, Lester L.  (2008) | The experience of pay for performance in English family practice: a qualitative study. | Conducted an in-depth exploration of family physicians’ and nurses’ beliefs and concerns about changes to the family health care service as a result of the new pay-for-performance scheme in the United Kingdom (Quality and Outcomes Framework [QOF]). | Qualitative Study | Semi structured interviews. | 22 nationally representative practices across England.  21 family doctors and  20 nurses. | The QOF scheme may have achieved its declared objectives of improving disease-specific processes of patient care through the achievement of clinical and organizational targets and  Increased physician income, but our findings suggest that it has changed the dynamic between doctors and nurses and the nature of the practitioner-patient consultation. |
| Lester H, Matharu T, Mohammed MA, Lester D, Foskett-Tharby R. (2013) | Implementation of pay for performance in primary care: a qualitative study 8 years after introduction. | To obtain a longer term perspective on the implementation of the QOF. | Qualitative study  Inductive coding that allowed themes to emerge from the data. | Semi-structured interviews. | 23 practices across England.  47 health professionals,  26 GPs (both profit sharing and salaried)  13 practice managers  6 practice nurses  2 practice administrative staff. | Pay for performance indicators are now welcomed by primary healthcare teams and GPs across generations.  Almost all interviewees wanted to see a greater emphasis on involving front line practice teams in developing indicators.  However, almost all GPs and practice managers described a sense of decreased clinical autonomy and loss of professionalism.  Calibrating the appropriate level of clinical autonomy is critical if pay for performance schemes are to have maximal impact on patient care. |
| Sudeh Cheraghi-Sohi S, Calnan M. (2013) | Discretion or discretions? Delineating professional discretion: the case of English medical practice | This paper attempts to fill this gap by delineating the key concepts of professional discretion evident in the literature and exploring their significance in an empirical study of the influence of the 2004 new general medical services contract (nGMS) and the introduction of the Quality and Outcomes Framework (QOF). | Longitudinal qualitative design. | Semi-structured interviews. | 33 different practices  62 general practitioners,  41 GP principals and  21 salaried GPs. | The findings suggest that through a complex interplay of factors, a post-QOF reduction in GP discretion was identifiable, highlighting different potential sources of constraint such as in the social, organisational and economic dimensions of discretion.  The evidence also suggested the emergence of a new form of organisational medical professionalism within general practice characterised by standardisation, bureaucracy and performance management. |
| NHS England | Report of the Review of the Quality and Outcomes Framework in England | The search aimed to identify published peer-reviewed empirical research relating to pay-for performance schemes in primary care in high income countries, focusing particularly on QOF. Only English language publications were sought. | Qualitative synthesis  Conducted a  comprehensive search for quantitative studies. We synthesised these qualitatively because the  studies had heterogeneous methods and outcomes so were not suitable for quantitative synthesis.  Also identified studies that included data relating to the three  supplementary subject areas (exception reporting, qualitative data and pay-for-performance in  other countries) and summarised these. | 40 studies included.  Also included:  Interviews.  Reference groups with patients and the public.  Reference groups with practice staff  Reference groups with commissioners.  Event for charities.  Conversations with innovators. | 7 qualitative studies of QOF.    23 studies of  effectiveness of QOF (6 systematic reviews, 9 longitudinal studies, 8 cross-sectional studies).  4 studies of variations in exception reporting in QOF.  7 studies of pay-for performance in other countries. | Qualitative studies of QOF  Researchers in the study of primary care staff’s views eight years after implementation of QOF  organised their findings, around three themes:  Routinisation of QOF into primary care work  Impact of QOF on medical professionalism.  Evolution of QOF in a primary care setting.  The study of ways which QOF had influenced the nature of medical professionalism, in particular the concept of clinical discretion, categorised these as:  Bureaucratic  Social  Organisational  Economic  Political  The studies of case-finding for depression found obstacles to appropriate screening for depression,  which could lead to a systematic under-detection. |
| McDonald M, Harrison S Checkland K. (2008) | Incentives and control in primary health care: findings from English pay-for-performance case studies. | The authors’ aimed to investigate mechanisms and perceptions of control following the implementation of a new “pay-for-performance” contract (the new General Medical Services, or GMS, contract) in general practice. | This paper was based on an in-depth qualitative case study approach.  Convenience sample. | Formal one to one interviews  Observational components involved clinics, GP and nurse consultations, office and reception area working patterns and practice meetings.  Informal conversations and interviews were conducted with all staff working in the practice (detailed field notes). | In 2 general practices in England.  12 GPs (2 salaried)  9 nurses,  4 healthcare assistants,  1 practice manager,  1 senior manager,  1 senior receptionist in each practice. | A distinction is emerging amongst ostensibly equal partners between those general practitioners conducting and those subject to surveillance.  Attitudes towards the contract were largely positive, although discontent was higher in the practice which employed a more intensive surveillance regime and greater amongst nurses than doctors. |
