# Supplementary material for: A pay for performance scheme in primary care: Meta-synthesis of qualitative studies on the provider experiences of the quality and outcomes framework in the UK
Source: BMC Fam Pract. 2020 Jul 13;21:142. doi: 10.1186/s12875-020-01208-8 (PMC7359468; doi:10.1186/s12875-020-01208-8)
Supplement: Supplementary file 3 — Additional file 3. Table. 5 First and Second Order Constructs from the Published Papers [file 12875_2020_1208_MOESM3_ESM.docx]

**Additional file 3**

**Table. 5 First and Second Order Constructs from the Published Papers**

| **First order constructs (Quotes from constructs)** | **Second order constructs from the papers** | **Extracted Constructs** |
| --- | --- | --- |
| It is more likely that nurses could manage without family physicians than we could manage without nurses (General practitioner [GP]8).  There’s a little bit of deskilling there. I mean, we have a respiratory nurse, and so she sees all the asthmatics and does all the routine checks on them.… But a lot of the asthmatics tend not (to see a GP) which is a good thing, because it means they’ll be better controlled. But on the other hand, I feel like, “Oh, an asthmatic patient! What do I do?” you know. ”Oh, I’ll send them to X,” you know. It’s like, that was a thing that I would have dealt with on my own before. But now it is better (GP10).  One doctor described this additional money as like “having 5 year’s pay rise in one go.” Some doctors believed they had already been working to a high standard, and the extra money was an acknowledgment of this previously unpaid work. And I think practices that were doing well anyway are still doing stuff above and beyond what’s in the new contract, as they always have, and are perhaps now getting rewarded for work that they were doing (GP4).  Well it’s certainly improved my income. Probably increased my workload, not to the same degree as it increased my income. But I’m a bit worried that we’ve sold our soul to the devil to some degree, because they can change the goal posts later (GP39).  I don’t think the £250,000 (media) headlines help much. I had an interesting discussion last week with a young man, a man my age—a 36-year-old guy—who was having a hard time of it at work, and he was talking about stress, and I said, “Oh yes, tell me about it,” and he said, “Och, stress, look at this, this is a cushy number you’ve got … you are getting plenty of money for it” (GP49).  I’m sure the doctors appreciate what the nurses do, but I’m sure that we haven’t had our salary updated as much as we should, for the money that they’re probably getting from QOF. And that’s, all 3 of us agree that. All the 3 nurses, we agree that we’re doing a lot more of their work for them, and not much in the way of money recognition, yeah (Practice nurse [PN] 47).  In the sense that it’s still a patient presenting to a doctor with a problem, yes, it is the same as it always was. The difference is that it’s more likely that the patient and the doctor won’t know each other (GP25). … with the asthma, the patients are beginning to see the same nurse, you know, rather than a different GP.… I will see the diabetics, and they know that I’ve been trying to say to them, “can you come, you know you can always come back,” and I always try and make it so that there is open access for them if they have got a problem (PN49). | **Effects of Implementing a Pay-for-Performance Scheme**  Most physicians believed that the quality targets had improved patient care by focusing attention on necessary clinical activities that might have been being neglected. Moreover, the targets had prompted staff to contact patients who were reluctant to attend, encouraged preventive work and chronic disease management, and promoted a sense of pride in achievement. Most doctors and nurses acknowledged that nurses had become the primary provider of health care for patients with chronic conditions.  Although most doctors welcomed the added value of this role, some doctors believed they had become deskilled in areas previously seen as a core part of their role.  All interviewees recognized that QOF had led to considerable extra income at the practice level. Although the new contract is a practice contract, and targets had been achieved by teams of doctors, nurses, and administrative staff, most of additional money became part of the doctors’ income as employers and owners of practices, regardless of the nurses’ contribution to achieving the targets.  The financial reward in return for extra work was also seen as helpful in raising morale within the profession and improving physician work-life balance. A minority of doctors, however, expressed concern about losing future control of patient care to the government because of, or perceived negative public opinion generated by, the increased family doctor income.  That only a small financial bonus had been paid to nursing staff in some (but not all) practices created a sense of resentment among a considerable minority of the nurses. These nurses perceived their increased autonomy, hard work, and chronic disease management roles as critical in achieving QOF targets.  Continuity of care was claimed as a central feature of both doctor and practice nurse roles.  For most nurses, interpersonal continuity was described as a relatively new feature as they assumed responsibility for patients with chronic conditions.  Doctors were far more likely to stress the importance of their longitudinal relationship with patients, but some also stressed that the contract had helped to accelerate a pre-existing decline in interpersonal continuity of care. | Most physicians believed that the quality targets had improved patient care by focusing attention on necessary clinical activities that might have been being neglected.  The targets had prompted staff to contact patients who were reluctant to attend, encouraged preventive work and chronic disease management, and promoted a sense of pride in achievement.  Most doctors and nurses acknowledged that nurses had become the primary provider of health care for patients with chronic conditions.  QOF had led to considerable extra income at the practice level.  Although the new contract is a practice contract, and targets had  It had been achieved by teams of doctors, nurses, and administrative staff, most of additional money became part of the doctors’ income as employers and owners of practices, regardless of the nurses’ contribution to achieving the targets.  The financial reward in return for extra work was also seen as helpful in raising morale within the profession and improving physician work-life balance.  A minority of doctors, however, expressed concern about losing future control of patient care to the government because of, or perceived negative public opinion generated by, the increased family doctor income.  That only a small financial bonus had been paid to nursing staff in some (but not all) practices created a sense of resentment among a considerable minority of the nurses.  These nurses perceived their increased autonomy, hard work, and chronic disease management roles as critical in achieving QOF targets.  **Continuity of care**  Continuity of care was claimed as a central feature of both doctor and practice nurse roles.  For most nurses, interpersonal continuity was described as a relatively new feature as they assumed responsibility for patients with chronic conditions.  Doctors were far more likely to stress the importance of their longitudinal relationship with patients, but some also stressed that the contract had helped to accelerate a pre-existing decline in interpersonal continuity of care. |
| In some respects my role hasn’t changed and never will do, as far as I can see, and not in my lifetime anyway. A person comes in the door, sits down and I ask them what’s wrong and you try and fi x it. That hasn’t changed (GP21).  Although not raised directly by interviewers, many participants used the concept and the term agenda spontaneously when describing the consultation. … so it’s made the 2 agendas a little bit clearer, and I guess you’ve always had a health agenda, which, I said before, if you come with a cold, your agenda and mine is probably never been the same, but now that mine is encapsulated by QOF … it’s a bit more blatantly not the same. So I think there is an intrusion there, and it’s not an entirely patient-led agenda, because you’ve got things that you want to do that you think are more important (GP2).  So there is all this going on that he, the patient’s agenda is one thing and yours is another … your brain’s working twice isn’t it, at the same time (GP38).  … in terms of asking them additional questions to fi t with QOF information that is required, I think that has altered, yes … at times I feel uncomfortable with consultations where I have to include certain things related to that that I might not normally have deemed relevant at that particular point in time (GP52).  And there have been 1 or 2 occasions where I went through the cholesterol, the depression, the CHD, and everything else, and “Oh, that’s wonderful, I’m finished now,” and the patient said “Well, what about my foot then?” “What foot” (GP39)? Some doctors and nurses stated they had made a point of apologizing or explaining to a patient why they were focusing on the targets as well as the patient’s issues. I feel actually I’m looking at the patient less than I used to, which is a shame.… I have to say to them, “I’m sorry, I’ve got to look at the computer as well and type in while you’re talking to me” (PN38).  Most doctors also stated that they would not pursue a target incentive if it might be detrimental to the patient. If their [HbA1c] is 8, better leave them there because the quality of life is good, instead of giving them high doses and all that, and getting it lower (GP7).  I tend to deal with the problem patients come with first. And then if it’s appropriate to ask questions, you know, ticking the boxes, I will do at the end of the consultation (GP10).  I suppose that I, I have less time to attend to social issues and unhappy patients. I tend to try and be sympathetic but within a shorter time period (GP20).  So maybe other data is not sort of recorded quite as well. There’s never as much training and education available for non-QOF areas as there are for QOF areas, so you don’t get the same updating, the same perhaps advice about managing those area (GP1). | **Consultation Process and Agendas**  All doctors reported that there had been no change to the essence of the face-to-face doctor-patient interaction within routine consultations, which are booked at 10-minute intervals.  Addressing a number of agendas within a single consultation was seen by many doctors as a key skill of good family practice and reflected a long-standing acknowledgment that a consultation could include acute and chronic problems, health promotion, and health prevention advice as appropriate to that consultation.  The context and consequences of the doctor patient interaction, however, were perceived by all doctors as having changed as a direct result of the pay-for-performance scheme. All participants acknowledged that the QOF had influenced their agenda.  The use of computerized chronic disease templates in consultations had been part of primary care in the United Kingdom for some years.  Although many doctors and nurses believed the templates played a positive role in prompting them to take action, such as measuring blood pressure, others believed the templates introduced a new element into the consultation and threatened to shift the balance away from the patient’s immediate agenda.  Some doctors commented on how pursuing QOF targets within a consultation could create a potentially conflicting doctor and patient dual agenda. Others described incorporating the QOF agenda while attempting to deal with the patient’s agenda, despite having reservations about the value of QOF work, whereas still others stated that this double agenda added to the workload of consultations and risked doctors being distracted from the patient’s agenda.  All were keen, however, to emphasize that the patient’s agenda came first and that QOF reminders flashed up on the computer would be bypassed if there was in sufficient time to address both.  There was, however, an interesting tension for a considerable minority of doctors. These physicians described how the generally accepted desire to meet target levels and provide better chronic disease management also meant that at times they felt they might be too proactive in following up patients to meet targets or attend appointments, reflecting a physician-centred, rather than a patient-centred, approach to care.  There was no consensus about whether addressing targeted areas had led to neglecting those areas within the consultation that are not incentivized.  Whereas most doctors believed that they treated all patients and patient conditions equally, a minority wondered whether there had been a subtle downgrading of conditions not incentivized in the QOF, as well as the social aspect of family medicine, through changes to available education and increased time pressures within consultations. | Change to the essence of the face-to-face doctor-patient interaction within routine consultations, which are booked at 10-minute intervals.  Addressing a number of agendas within a single consultation was seen by many doctors as a key skill of good family practice and reflected a long-standing acknowledgment that a consultation could include acute and chronic problems, health promotion, and health prevention advice as appropriate to that consultation. The context and consequences of the doctor patient interaction, however, were perceived by all doctors as having changed as a direct result of the pay-for-performance scheme. All participants acknowledged that the QOF had influenced their agenda.  The use of computerized chronic disease templates in consultations had been part of primary care in the United Kingdom for some years.  Although many doctors and nurses believed the templates played a positive role in prompting them to take action, such as measuring blood pressure, others believed the templates introduced a new element into the consultation and threatened to shift the balance away from the patient’s immediate agenda.  Some doctors commented on how pursuing QOF targets within a consultation could create a potentially conflicting doctor and patient dual agenda.  Others described incorporating the QOF agenda while attempting to deal with the patient’s agenda, despite having reservations about the value of QOF work, whereas still others stated that this double agenda added to the workload of consultations and risked doctors being distracted from the patient’s agenda.  All were keen, however, to emphasize that the patient’s agenda came first and that QOF reminders flashed up on the computer would be bypassed if there was in sufficient time to address both.  There was, however, an interesting tension for a considerable minority of doctors. These physicians described how the generally accepted desire to meet target levels and provide better chronic disease management also meant that at times they felt they might be too proactive in following up patients to meet targets or attend appointments, reflecting a physician-centred, rather than a patient-centred, approach to care.  **Not incentivized areas**  There was no consensus about whether addressing targeted areas had led to neglecting those areas within the consultation that are not incentivized.  Whereas most doctors believed that they treated all patients and patient conditions equally, a minority wondered whether there had been a subtle downgrading of conditions not incentivized in the QOF, as well as the social aspect of family medicine, through changes to available education and increased time pressures within consultations. |
| My honest feeling is that actually nobody has a grand plan, or that there may be several different grand plans which actually don’t interrelate with each other, and that people have started initiatives on the basis of one idea and then another initiative on the basis of another idea, and there isn’t actually a grand plan (GP49).  There is an environment and ethos of increased surveillance and performance monitoring (GP1). I suppose it feels more like I’m being watched. It’s a little bit like big brother—you’ve not ticked these boxes (PN2). There was general agreement that irrespective of the physician’s own personal views of the relevance and legitimacy of targets, physicians felt motivated to achieve the highest achievement rate possible for their practice and their own income. I think that GPs, and doctors by nature, are competitive, and so one wants to get all the brownie points that one can … (GP20).  The workload has gone up, but then there’s more reward if we achieve certain goals, the reward is there. If you don’t achieve the reward, you don’t get it (GP32).  … the ones where you seem to be collecting data purely for the fact of collecting the data. So I mean initially, for instance, when the renal targets came in and things, it was a case of have a register of people and what stage of chronic kidney disease they were. But there was very little guidance as to what we were supposed to do with them having identified that (GP52).  You know that depression screening ones, because I think someone comes to you, you do a check on them, you immediately, you’ve known them for a long time and then you think, ”Oh, I’ve got to ask those 2 questions.” You get these questions and you feel a bit of a fool because you, you just know by common sense how they are so I don’t think that was a great thing to put in (N25). | **Performance Monitoring and Competition**  The interviews highlighted a sense of underlying uncertainty about the future of family medicine in the United Kingdom. Many doctors were worried that there was “no grand plan” within the NHS underpinning the raft of recent reforms. This uncertainty was further aggravated by a negative perception that the new targets formed part of an ever-increasing performance-monitoring and surveillance culture by government agencies.  **New Indicators Introduced in 2006**  All doctors and nurses stated that they could see the purpose of most of the indicators in the 2004 iteration of the QOF. More concern was expressed about the relevance to patients of, and work required for achieving, targets introduced in the revised QOF in 2006.  These new indicators relate to setting up registers of patients with newly diagnosed chronic kidney disease and obesity and measuring the severity of depression in people with a new diagnosis, which one doctor referred to as “not a family practice way of doing things.”  No interviewee challenged the importance of these issues or stated their intention not to attempt to meet the targets. A minority of doctors and nurses, however, queried whether these targets were aligned with clear primary health professional objectives and were a core part of family practice and whether they would result in improved patient outcomes. | The interviews highlighted a sense of underlying uncertainty about the future of family medicine in the United Kingdom. Many doctors were worried that there was “no grand plan” within the NHS underpinning the raft of recent reforms. This uncertainty was further aggravated by a negative perception that the new targets formed part of an ever-increasing performance-monitoring and surveillance culture by government agencies.  All doctors and nurses stated that they could see the purpose of most of the indicators in the 2004 iteration of the QOF  More concern was expressed about the relevance to patients of, and work required for achieving, targets introduced in the revised QOF in 2006  These new indicators relate to setting up registers of patients with newly diagnosed chronic kidney disease and obesity and measuring the severity of depression in people with a new diagnosis, which one doctor referred to as “not a family practice way of doing things.”  No interviewee challenged the importance of these issues or stated their intention not to attempt to meet the targets. |
