# Supplementary material for: A pay for performance scheme in primary care: Meta-synthesis of qualitative studies on the provider experiences of the quality and outcomes framework in the UK
Source: BMC Fam Pract. 2020 Jul 13;21:142. doi: 10.1186/s12875-020-01208-8 (PMC7359468; doi:10.1186/s12875-020-01208-8)
Supplement: Supplementary file 4 — Additional file 4. Table. 6 Identifying Third Order Constructs [file 12875_2020_1208_MOESM4_ESM.docx]

**Additional file 4**

**Table. 6 Identifying Third Order Constructs**

| **Concepts from the studies in the review** | **Reconstructing the findings** | **Themes for the synthesis** |  |
| --- | --- | --- | --- |
| Practices had made extensive alterations to their organization in terms of staff appointments, skill mix, computer coding and appointment systems in order to more actively identify and recall patients, agree practice wide protocols in accordance with the QOF, deliver standardized care as a team, and consistently code care processes (Maisey et al 2008).  Whilst the details were different in each practice, in general this involved an increase in the number of administrative staff, including those with responsibility for information technology (IT) (Checkland & Harrison 2010).  This new managerial stratum actively worked to align their colleagues’ clinical activities to the wider organisational goals (Cheraghi-Sohi & Calnan 2013).  Changes resulting from introduction of the QOF on practice organisation, lead to an increased role for information technology (IT) (Checkland K, Harrison 2010).  It is important to consider retaining aspects of QOF that may deliver benefits; for example, GPs report (anecdotally) that they find the electronic prompts to deliver care of long term conditions useful, so that they can be sure that certain issues have been dealt with without having to search through case-notes NHS England 2018).  Most English general practices have reached the upper payment thresholds for QOF and therefore maximum remuneration. The implication of this ‘ceiling effect’ is that practices may not have been strongly motivated to increase their achievement further, for example, achieving the targets in those who have the complex and difficult-to-manage problems, but who are not excepted (NHS England 2018)  No definitive evidence found to inform what would happen to performance on QOF indicators were the financial incentive removed, and no evidence to inform us what would happen to quality of primary care – although it is unlikely that this would be significant given the narrow view of quality that the QOF embodies (NHS England 2018).  Were the QOF to be abolished, it important to remember, though, that it provides a major component of practice income; practices must be protected from loss of income, which would almost certainly have a detrimental effect on patient care and further worsen recruitment and retention in primary care (NHS England 2018).  Almost all interviewees wanted to see a greater emphasis on involving front line practice teams in developing indicators. (Lester H et al 2013, NHS England).  For some of the more ambitious outcome indicators, for example, measures of strict blood glucose control of diabetes, it may be that practices are not motivated to achieve upper thresholds because the role of patient compliance is so strong that they are seen as unachievable (NHS England 2018)  Professionals appeared more preoccupied by their lack of control in achieving indicator targets, especially if dependent upon patient cooperation (Hacket et al 2014). Limited availability of appropriate, supportive resources needed to address such problems further undermined confidence in these targets (Hacket et al 2014).  Most QOF indicators, however, measure activities that are about single dimensions of primary care (NHS England 2018). | **Policy-organizational:**    Were characterised by a reformation of practice organisational structures and what constituted teamwork.  The advancement of IT was experienced in two ways. As a tight but helpful tool to remember, manage, collect relevant patient illness related data and on the other hand to make visible the performance of professional work against outsider implemented targets.  Extensive improvement was seen as an effect of consistency and recording of incentivized conditions, new protocols linked to wider Governmental goals.  There seemed to be insufficient Governmental, organisational, administrative, executive, and managerial recognition of the link between the ‘doctors on the ground floor’ working without doubt in ‘everyday clinical practice’, and a realisation of the consequence for ‘ routine clinical practice’ and for the professionals’ and patients’ concerned, who indeed make up the influential part of this balance. | ***Structural & Organizational changes:***   \| Increased staff appointments  Computer coding  appointment systems for  ways of identifying patients  Practice wide protocols  Deliver standardised care   Increased number of staff for IT  ***Information Technology:***  Evolving role of IT  Used as surveillance of targets being met  Mechanism in place for following the work  of others,  More business orientated structure,  performing against targets  Prompts from IT as reminders \| \| --- \|   ***Ownership & Appropriating Indicators:***  Understandable reasons for most of the indicators  Clinical indicators not exclusively handled initially by clinical staff  No discussion on the practicality of the indicators to clinical practice occurred  Were indicators a technical problem or clinical  Should involve frontline clinical teams in developing indicators  Issues of control with achieving targets, especially those dependent on patient cooperation | **Structural changes**  **Structural changes**  **Loss of autonomy and control** |
| The introduction of the contract has allowed this GP to exercise a “hands-on” approach to a much greater degree than was possible previously (McDonald Harrison Checkland 2008).  The new GMS contract has given practice nurses increased responsibility. (McGregor et al 2008). Nurses experienced increased workload but enjoyed more autonomy and job satisfaction (Maisey et al 2008)  The QOF has implications for the primary care workforce.75 Since 2004, practice nurses carry out far more consultations – an increase from 21% to 35% of general practice consultations, 76 and there have been changes to professional boundaries.77 Many routine tasks have passed from GPs to practice nurses or health care assistants, and there has been reorganisation of care into chronic disease clinics. Practice staff to carry out the information technology tasks needed to collate data for QOF have also increased (NHS England 2018)  Case findings often occurred within tightly structured and time-limited chronic illness reviews required to document QOF processes of care, and appeared to exacerbate existing discordance. This led to professionals disregarding attempts by patients to steer the consultation around to their own perceived needs (Alderson et al 2014; Maxwell et al 2013, NHS England).  Participants’ accounts of the contract and QOF appeared to change over time. The initial 2004 version was deemed to have been largely positive and beneficial both to the profession and patients.  GPs spoke of how the initial version had provided a measure of the output of general practice which had to that point been ‘invisible’ and under-valued: (Cheraghi-Sohi & Calnan 2013, NHS England)  NHSE Found some evidence that QOF may have positive effects on some processes of care. However, the evidence suggests that the effect is short-lived for most of these. We found no evidence that QOF is reducing inequalities in processes of care or outcomes that were incentivised by QOF (NHS England 2018).  Found no evidence of the effects of QOF on other aspects of primary care, specifically on the delivery of holistic care, continuity of care, integrated care or patient-centred care – those elements of primary care that are prioritised in the Five Year Forward View. In fact, research to date has not attempted to identify the effects on these outcomes, having examined effects only on easily measurable outcomes, for example those collected as part of QOF, or routinely available data on mortality, emergency admissions, consultation rates, and prescribing (NHS England 2018)  NHSE found that the evidence that QOF improves health care quality is limited. First, because QOF was implemented throughout the UK, there are no reliable controls in the studies. Second, while QOF has led to a lot of research attempting to evaluate its effect, what has been published to date tends to report its effect using performance on the QOF indicators themselves or other routinely available data as measures of quality (NHS England 2018).  No evidence that QOF encourages any other aspect of primary care performance than those elements incentivised by the QOF. Also found no evidence that QOF is an effective mechanism for reducing inequalities in health and health care. It may even worsen inequalities if patients in whom clinical objectives are more easily met are targeted by QOF activities rather than those with more complex health and social problems.  GPs voiced positive overall attitudes to the new contract (McDonald Harrison Checkland 2008). Some nurses felt that there was more of a focus on population health than on the needs of the individual. They were particularly frustrated by the rigid protocols for reviewing patients and the call and recall system (McGregor et al 2008).  Pay-for-performance schemes to retain, attract and motivate primary healthcare professional represent a narrow view of motivational and satisfaction mechanisms (NHS England 2018).  An evidence synthesis on GP recruitment and retention show that there is little evidence to support the idea that financial schemes increase satisfaction or reduce demotivation or dissatisfaction (NHS England 2018).  Permitted reasons for exception reporting include logistical considerations (e.g., recent registration of the patient with the practice), clinical reasons (e.g., the presence of a supervening condition or terminal illness) and patient-informed dissent (ie, not agreeing to the investigation or treatment). Recently diagnosed or registered patients are automatically excluded by clinical computing systems, whereas practices must actively identify patients who meet other exclusion criteria. (Lester, Hannon, Campbell 2011)  A few GPs spoke about how patients were considered for exception reporting as practice staff saw them throughout the year (whether at review or opportunistically), but most described a greater focus on exception reporting towards the end of the QOF financial year. For some, this was described as good practice in trying to make a clinical register as complete as possible but, for others, it was also an opportunity to see whether targets had been met and, if not, whether exception reporting should be considered for remaining patients to hit the target. (Campbell, Hannon, Lester, 2011).  In 2014/5, exception rates were less than 10% for most indicators. It is not possible to ascertain from the available data what the ‘correct’ exception rate is for each indicator, and in any case this will vary by practice because of differences in population makeup. However, the low use of exceptions suggests that what is known as ‘gaming’ (working particularly hard to identify patients as exceptions in order to maximise points earned and therefore income) was not common. Moreover, it appears that there are good reasons for high exception rates where they occur (NHS England)  For example, many intervention indicators had high exception rates because patients may decline to receive interventions more readily than investigations or clinical reviews. Also, we expected the observed high exception rates for some of the outcome indicators, because for many, patient compliance to lifestyle advice is critical and patients may actively decline to follow this (NHS England). | **Provider-level impacts:**  Lead doctors now carried greater responsibility in most areas of their practice.  Salaried GPs apparently carried less responsibility for QOF  The changes for nurses were perceived to be constructive on some fundamental areas  Nurses saw an increase in workload, autonomy and satisfaction with their role  Not sure if this lead to increase in status for nurses  Managerial roles were evolving with more defined responsibilities for QOF activity.  The tight structure and time limited nature of chronic illness reviews were not without a struggle. There was no scope for mutual concessions and compromises around perceived need.  The profile of General Practice was subsequently raised & valued at a much needed time for the profession. Neglected clinical activity was targeted, by quality targets and pressures of modern day practices were re-evaluated.  Overall aim of QOF as a mechanism for improving and/or standardising quality of care within practices was perceived as ‘better quality of care’ overall.  Care was systematised and standardised. Financial reward in return for extra work was linked to a raise in morale within the profession and improving physician work-life balance.  Acceptances of routine standardised care, behaviours were seen to come in line with the changes. Some frustration with elements that were repetitive and did not sit well with everyday practice, leading to unintended consequences.  QOF depended upon the practices -honest and ethical application in clinical practice.  Some areas of QOF were open to particularly high exception reporting and the possibility of gaming. These were identified, with possibly reducing the occurrence through piloting ahead of any acceptance & implementation. | ***Doctors***  Clinicians’ work appeared more hands on, more intense routines, extra-long working hours, supervising the work of nurse colleagues  Clinician dissatisfaction with increased business like role  Salaried clinicians appeared less engaged  lesser responsibility towards the business end of the professional role  ***Nurses & Allied Health care professionals***  Hierarchy still existed, with additional new roles or QOF teams to achieve QOF targets  There were rewards for engaging with QOF but not everyone was rewarded financially in this engagement with QOF  Collective effort with collective target  Banner of practice staff now included not just clinicians and nurses but also receptionist, IT teams and those with managerial responsibilities  ***Case finding, Screening & Evidence Based Medicine (EBM):***  Tightly structured reviews  Time limited illness reviews  No time for the patient to be heard  There was a difference for nurses in an encounter that required obtaining something that could be measured to something more subjective  ***Improvements:***  Original QOF changes were perceived positively & beneficial  QOF gifted an increase in   - status for general practice - rewarded the profession - recognised the pressures - visibility to the clinical specialty - greater importance than ever before   ***Views, Attitude & Behaviour Change:***  Clinicians in general practice expressed a positive attitude.  Nursing colleagues saw a more of a focus on population health than the individual.  Nurses upset by rigid protocols for reviewing patients, call and recall.  Freedom to deliver quality services was wiped out by a system involving greater surveillance and scrutiny of performance  ***Fair play & Exception Reporting:***  QOF was applied honestly and ethically by clinicians at their preference  Within the context of the pilot, indicators were identified for which there might be particularly high exception reporting and therefore the possibility of gaming | **Incentivised conformity**  **Control and ownership**  **Continuity of care – Nurses**  **Conformity – not incentivised by monies**  **Loss of autonomy, holism and control**  **Systemised and standardised care**  **Loss of autonomy and control**  **Complex care** |
| The context and consequences of the doctor patient interaction, however, were perceived by all doctors as having changed as a direct result of the pay-for-performance scheme. All participants acknowledged that the QOF had influenced their agenda (Campbell et al 2008 Cheraghi-Sohi & Calnan 2013).  Moreover, participants acknowledged that their consultations had become more ‘biomedical’, with an additional QOF-related agenda running alongside the patient’s own agenda. (Checkland & Harrison 2010).  Case 1 - there was a missed opportunity for the PN to explore the patient’s knowledge and understanding of COPD, its management, and the place of nebulizers in the consultation. The PN, by not exploring the patient’s knowledge and concerns, and not conveying her thinking about the possible use of nebulizers, caused confusion for the patient, and left him with a sense that nothing can be done to help him (Chew-Graham et al 2013).  Previously, the practice had assumed that it was performing well, since complaints were low, patients appeared happy and staffs were given a large degree of freedom when deciding on the appropriate way to deliver services (McDonald Harrison Checkland 2008).  Doctors, especially, expressed concerns about loss of holistic care and a skewing of effort towards incentivized activities (Maisey et al 2008).  All were keen; however, to emphasize that the patient’s agenda came first and that QOF reminders flashed up on the computer would be bypassed if there was insufficient time to address both (Campbell et al 2008).  We found no evidence that QOF encourages any other aspect of primary care performance than those elements incentivised by the QOF. In particular, it does not reward holistic care, integrated care or patient-centred care. Therefore, there is no evidence that QOF will advance progress towards the aims of the Five Year Forward View significantly. QOF encourages a narrow, biomedical view of health care performance, and ‘high performance’ does not necessarily mean ‘high quality’ (NHS England 2018).  Although participants emphasised the importance of traditional general practice values, such as holism and continuity, the majority felt that the 2004 changes had impacted on these values. (Cheraghi-Sohi et al 2012).  Participants related that patients now experienced less continuity with their GPs. Participants highlighted two major contributing factors to this: the role of practice nurses in conducting the day-to-day QOF template work, and the impact of opting out of providing out-of-hours care. While the changes meant individual GPs had increased flexibility in terms of work–life balance, they often perceived that patient care had declined as a result (Cheraghi-Sohi et al 2012).  Most participants however described responding flexibly in an attempt to maintain espoused preferred and/ or prior ways of consulting which they invariably described as patient-centred (Cheraghi-Sohi & Calnan 2013; McDonald Harrison Checkland 2008).  A substantial minority considered standardized care to be a ‘box-ticking’ exercise, felt their new role to be at odds with their professional training as generalist doctors and saw their caring role as unrecognized in the contract. Some of these respondents described the need to defend efforts to continue to deliver non-incentivized care as part of their professional role (Maisey et al 2008).  The evidence suggests that QOF may have positive and negative effects on motivation for primary care professionals and this highlights the need to consider both intrinsic and extrinsic motivational factors, and among extrinsic factors, to consider non-financial rewards (NHS England 2018).  Motivation to deliver high quality care among health professionals is complex, but it is likely that other motivational factor than financial rewards may be effective. If the NHS is to deliver the aims of the Five Year Forward View, in the context of a demoralised primary care workforce, it is important to consider other measures of quality of primary care and other ways of motivating health professionals to deliver high quality care (NHS England 2018). | **Provider-patient relationship:**  There has been frequent change in the Health Services and with that came considerable discourse around consultations & much has been conceptualised academically, yet we still want to determine if ‘in the crux of it’ can equilibrium be achieved, between the QOF requirement or ‘any requirement from reform’ alongside a ‘persons’ perceived need, in a tightly structured, time limited consultation in ‘everyday clinical practice’.  Happy patients and low number of complaints were maybe the measure of good performance in the past.  Now the clinicians were propelled to change alongside QOF.  The pace of change was not only fast but firm towards incentivised activities. As a result clinicians were more proactive in their patients’ health. However this interest felt skewed for some. Patient care was felt to be more clinician- centred. This had some implications for responsibility of care; had QOF shifted this responsibility somewhat into the clinician’s domain. Patients were called and recalled for reviews, depending on their health and changes to the clinics and resulting structure of healthcare.  The two values that were seen to be linked to general practice and were particularly vulnerable to QOF changes; were holism and continuity of care. The caring role of clinician’ was not recognised in the QOF changes. Hence the QOF requirements were deemed the professional role. Also, being patient centred clinician was deemed a selective value, with some clinicians seen more or less patient centred.  Clinicians reported a ‘personal tussle’ with trying to hold on to old ways of working or trying to combine them with meeting QOF requirements. | ***Consultations***  The context and consequences of the doctor patient interaction changed  QOF influenced the clinicians agenda  Consultations were biomedical with an additional QOF-related agenda running alongside the patient’s own agenda.  ***Patient centred care***  Low level of patient complaints  Patients appeared happy  Clinicians’ had freedom on deciding on the appropriate delivery of care  ***Values***  holism and continuity seen as traditional general practice values impacted by QOF changes  patient continuity with clinicians declined due to   - increased nursing roles in QOF template work, - the impact of opting out of providing out-of-hours care   Attempts to conserve preferred/prior ways of consulting, that were patient centred  Standardized care to be a ‘box-ticking’ exercise, new role to be at odds with their professional training as generalist doctors  Caring role as unrecognised in the contract  Some described the need to defend efforts to continue to provide non-incentivized care as part of their professional role  Despite evidence of a more biomedical approach,  participants claimed that they still were able to practice ‘holistic’ medicine | **Holistic care**  **Patient centred care**  **Loss of holistic care**  **Effort focused towards incentivized activities**  **patients came first, this was reflected in the bypassing of QOF reminders**  **Holism and continuity of care** |
