# Supplementary material for: A pay for performance scheme in primary care: Meta-synthesis of qualitative studies on the provider experiences of the quality and outcomes framework in the UK
Source: BMC Fam Pract. 2020 Jul 13;21:142. doi: 10.1186/s12875-020-01208-8 (PMC7359468; doi:10.1186/s12875-020-01208-8)
Supplement: Supplementary file 5 — Additional file 5. Table. 7 Application of the Third Order Constructs to the Ten Motivational Values [file 12875_2020_1208_MOESM5_ESM.docx]

**Additional file 5**

**Table. 7 Application of the Third Order Constructs to the Ten Motivational Values**

| **Dimensions** | **Motivational Goal and Base Theory** | **Ten motivational Values** | **Third order constructs** |
| --- | --- | --- | --- |
| **Conservation** | The motivational goal of this value type is safety, harmony, and stability of  Society, of relationships, and of self.  It derives from basic individual and group requirements (cf. Kluckhohn, 195 1; Maslow, 1959; Williams, 1968) (social order, family security, national security, reciprocation of favors, clean, and sense of belonging, healthy). | **Security** –  Safety, harmony, and stability of society, of relationships, and of self.  National security  Reciprocation of favours  Family security  Sense of belonging  Social order  Healthy  Clean | EBM  Found that the evidence that QOF improves health care quality is limited. None of the research we found identified in the first instance a measure of high quality of care and used it to evaluate the QOF (NHS England 2018)  Almost every interviewee was positive about the impact of introducing pay for performance into primary care (Lester et al 2013). While some of this positive reaction was linked to comparisons with the state of primary care in other countries, most reflected comparisons with previous working practices and in particular, drew attention to the evidence-based nature of the indicators (Lester et al 2013). There was a retrospective sense of disbelief from GPs of all ages that individualistic non-uniform care between practices had been allowed to persist for so long (Lester et al 2013).  Participants’ accounts of the contract and QOF appeared to change over time. The initial 2004 version was deemed to have been largely positive and beneficial both to the profession and patients. GPs spoke of how the initial version had provided a measure of the output of general practice which had to that point been ‘invisible’ and under-valued: Cheraghi-Sohi & Calnan 2013  There was a sense of pride in practising evidence-based medicine (Lester et al 2013). GPs in the UK have, along with their hospital counterparts, been encouraged to engage with the notion of evidence-based medicine (EBM). The original proponents of this approach emphasised the integration of population evidence from randomised controlled trials with the unique personal preferences and health state of the individual in a way that is entirely compatible with a patient-centred approach (Checkland & Harrison 2010). The very nature of the QOF suggests a biomedical approach to medical practice and in their studies Checkland & Harrison found that changes had been made that would result in patients receiving a more biomedical, less patient-centred form of care. (Checkland & Harrison 2010). Furthermore, the importance of following rules or following evidence-based medicine (EBM) was replete throughout accounts and participants commonly perceived QOF to be largely evidence based, EBM had become viewed as the normative way of practising medicine (Cheraghi-Sohi & Calnan 2013).  Nurses were largely supportive of the contract and much more likely to comply with the lead partners’ wishes (McDonald Harrison Checkland 2008).  All doctors and nurses stated that they could see the purpose of most of the indicators in the 2004 iteration of the QOF (Campbell et al 2008).  The relatively rapid way in which pay for performance had become a routine part of primary care was also helped by the structure it gave to the practice year. GPs, practice managers, and nurses talked about the need to put more time aside to work on pay for performance indicator issues in the second half of each financial year, creating a QOF-related seasonal rhythm to workload from April to March (Lester et al 2013).  Size of practice and socioeconomic profile were not cited as facilitators or barriers to care, (Maisey et al 2008. Except that respondents from smaller practices in deprived areas felt under-resourced. Maisey et al 2008).  Many recognised that pay for performance indicators were traditionally best applied to ‘simple’ tasks such as achieving blood pressure targets, however, there was also a clear sense that in future, pay for performance could include indicators that challenged the practice team from an educational and organisational point of view (Lester et al 2013).  Although workload had increased, most nurses agreed that the contract had systematised and standardised care. Those in low-achieving practices were particularly likely to mention this (McGregor et al 2008). By contrast, high-achieving practices were more inclined to report that the contract represented an extension of previous ways of working (McGregor et al 2008). |
|  | The defining goal of this value type is restraint of actions, inclinations, and impulses likely to upset or harm others and violate social expectations or norms.  It is derived from the requirement that individuals inhibit inclinations that might be socially disruptive if interaction and group functioning are to run smoothly.  Conformity type values are mentioned in virtually all value analyses (e.g., Freud, 1930; Kohn & Schooler, 1983; Moms, 1956; Parsons, 1957).  Conformity values emphasize self-restraint in everyday interaction, usually with close others (obedient, self-discipline, politeness, honouring parents and elders). | **Conformity** –  Restraint of actions, inclinations, and impulses likely to upset or harm others and violate social expectations or norms.  Obedient  Self-discipline  Politeness  Honouring of others. | The findings suggest that QOF impacted on relationships within practices by placing a focus on the need for collective effort towards a collective organisational target (Cheraghi-Sohi & Calnan 2013).  On the basis of collective responsibility, the move towards a practice-based contract was seen as fundamental shift, particularly in an environment of increasingly mixed economies where salaried colleagues and other ancillary staff form an ever growing component of practice staff (Cheraghi-Sohi & Calnan 2013).  New clinical leads were identified for areas not previously covered, with the result that the nurses and GPs each had lead responsibility for one or more target areas (McDonald Harrison Checkland 2008).  There were changes in skill mix in the teams, with increased numbers of staff and delegation of duties (Maisey et al 2008). Respondents acknowledged longer term trends in devolution of care to nurses and fragmentation of care, but thought that these had been significantly accelerated by the need to meet the QOF requirements (Maisey et al 2008).  Participants described an increasingly antagonistic relationship between themselves, their professional negotiators and the state (Cheraghi-Sohi & Calnan 2013).  No practice had a formal, written practice policy on exception reporting, but most focused on two key principles. The first of these related to adherence to the external national guidance rules on (nondiscretionary) exception reporting19 as well as a parallel, internal, practice-specific focus reflecting on the suitability of individual patients for discretionary exclusion (Campbell, Hannon, Lester, 2011).  While some regarded the extra QOF earnings as a deserved pay rise, many GP principals reported investing initial QOF monies into their practices by making the types of organizational changes.  Subsequent changes to the contract however meant that the issue of finance became an increasingly key issue in participants’ beliefs about QOF and their adherence to QOF (Cheraghi-Sohi & Calnan 2013). They described how extra investment/outgoings, combined with a series of zero changes in financial ‘uplift,’ target thresholds being raised and a need to maintain income levels effectively left many perceiving that there was little room for them to disengage from any aspect of the contract (Cheraghi-Sohi & Calnan 2013)  The introduction of the contract has allowed this GP to exercise a “hands-on” approach to a much greater degree than was possible previously (McDonald Harrison Checkland 2008). Extra-long working hours lead partner was forced to work in the practice beyond their contracted hours and during annual leave (McDonald Harrison Checkland 2008).  Salaried GPs were often viewed by their principal counterparts as less engaged in QOF activity and this view was supported by a small proportion of salaried GPs who viewed the overall responsibility of meeting QOF targets as that of their other ‘managerially focused’ colleagues (Cheraghi-Sohi & Calnan 2013).  Salaried GP accounts suggested that economic issues were not seen as a direct constraining factor and cast themselves as largely removed from economic concerns which at times they viewed as affecting their principal’s approach to patient care (Cheraghi-Sohi & Calnan 2013).  The line ‘we’ve got no choice but to do it’ in terms of the financial implications characterized many participants’ sentiments (Cheraghi-Sohi & Calnan 2013). Despite such beliefs, a fractious point had not been reached; in fact the vast majority of GPs were seemingly striving to meet all of the new changes as they arose on an annual basis, irrespective of their views on the perceived motives behind the changes. Their motivation to do this however was seemingly based on a combination of the other factors (Cheraghi-Sohi & Calnan 2013). The targets had prompted staff to contact patients who were reluctant to attend, encouraged preventive work and chronic disease management, and promoted a sense of pride in achievement (Campbell et al 2008). Finally, it was clear that care within our practices had become more dependent upon pharmacological approaches to treatment, as the QOF requires blood pressures, for example, to be controlled within a certain period of time after diagnosis. Non-pharmacological measures may take time to work, and we found an increased tendency to treat early with tablets (Checkland & Harrison 2010).  Patients’ concerns appeared to have become a relatively low priority in the consultation, compared to the clinician-led agenda of the QOF: many respondents felt a pressure to concentrate on incentivized standards in the consultation, at the expense of other aspects of care. Maisey et al 2008  Most participants described a continuing principle of sharing clinical decisions with patients at an individual level, but a few acknowledged pressures to emphasize issues incentivized by the contract. Maisey et al 2008  A substantial minority considered standardized care to be a ‘box-ticking’ exercise, felt their new role to be at odds with their professional training as generalist doctors and saw their caring role as unrecognized in the contract. Some of these respondents described the need to defend efforts to continue to deliver non-incentivized care as part of their professional role. Maisey et al 2008  A minority of doctors, however, expressed concern about losing future control of patient care to the government because of, or perceived negative public opinion generated by, the increased family doctor income. Campbell et al 2008  Continuity of care was claimed as a central feature of both doctor and practice nurse roles. Campbell et al 2008  For most nurses, interpersonal continuity was described as a relatively new feature as they assumed responsibility for patients with chronic conditions. Campbell et al 2008  Doctors were far more likely to stress the importance of their longitudinal relationship with patients, but some also stressed that the contract had helped to accelerate a pre-existing decline in interpersonal continuity of care. Campbell et al 2008 |
|  | Groups everywhere develop symbols and practices that represent their shared experience and fate. These eventually become sanctioned as traditions and customs that are valued by the group’s members (Sumner, 1906).  Traditional  modes of behaviour become symbols of the group’s solidarity, expressions of its  unique worth and presumed guarantors of its survival (Durkheim, 1912/1954; Parsons, 1957).  Traditions most often take the form of religious rites, beliefs, and norms of behaviour (Radcliffe-Brown, 1952). The motivational goal of tradition values is respect, commitment, and acceptance of the customs and ideas that  one’s culture or religion impose on the individual (respect for tradition, humble, devout, accepting my portion in life, moderate). | **Tradition** –  Respect, commitment, and acceptance of the customs and ideas that one's culture or religion provides.  Respect for tradition.  Devout  Accepting my portion in life  Humble  Moderate | In all practices, data recording via templates had become the norm. These templates act both to define the nature of the work required by acting as ‘prompts’, and to discourage staff from recording uncoded information that is not important for the QOF process (Checkland & Harrison 2010).  Not only will a structured record shape the way a job is performed from day to day, but it becomes part of the definition of the nature of that job in the longer term. (Checkland & Harrison 2010).  The application of expertise in the service of others was clearly recognised and reinforced through the use of evidence based indicators (Lester et al 2013).  One-quarter of GPs described it as ‘disheartening’ to have indicators removed and wanted to continue to be paid a small amount of money to reflect the ongoing workload ‘something for maintaining quality you know’. So although a financial penalty, through the removal of process measures, and work load penalty, through the increased work needed to achieve new indicators in new clinical areas, appeared acceptable to a majority of GPs, a sizeable minority felt it was unfair (Lester et al 2013).  Whilst one GP outlined how his beliefs about work-life balance have been at odds with those of his partners for some time, he describes support for the system and not having to “be here in the evenings and the weekends, adding up numbers” and by implication, leaving others to do that. This suggests that what might previously have been a debate about practice philosophy, with little impact on the working patterns of individual GPs, has been transformed into something more concrete, which impacts on partners’ working practice, as a result of QOF and the policies adopted to ensure its implementation (McDonald Harrison Checkland 2008).  GP, described by a colleague as “more person centred”, early on in the project had expressed support for the new way or working. The above comments were made after we observed him in consultations making notes on paper and entering the information into the computer when the last patient had left the consulting room. McDonald Harrison Checkland 2008  Most participants however described responding flexibly in an attempt to maintain espoused preferred and/ or prior ways of consulting which they invariably described as patient-centred. Cheraghi-Sohi & Calnan 2013. |
| **Self enhancement** | This value type, derived from organismic needs and the pleasure associated with satisfying them, is referred to by scholars from many disciplines (e.g., Bentham, 1938-1948; Freud, 1933; Moms, 1956; Williams, 1968). It was formerly called “enjoyment” in order to justify including two values from the  Rokeach list, happiness and cheerful.  Neither of these values is convincingly derived from organismic needs. The  motivational goal of this type more sharply as pleasure or sensuous gratification for oneself (pleasure, enjoying life). | **Hedonism** –  Pleasure or deep gratification for oneself.  Enjoying life | One common view was that the changes to the contract and QOF were a timely and much needed change, which rewarded their profession and recognized the pressures associated with modern general practice (Cheraghi-Sohi & Calnan 2013). General practice has therefore assumed a greater importance than ever before (Checkland & Harrison 2010).  The financial reward in return for extra work was also seen as helpful in raising morale within the profession and improving physician work-life balance (Campbell et al 2008). Although salary increases, while remaining on the same grade, were unusual, approximately a third of interviewed nurses had been promoted to a higher grade since the inception of the new GMS contract — which would, of course, improve salaries. This appeared to be associated with higher-achieving QOF practices, but it was unclear if these promotions were as a direct result of QOF achievement (McGregor et al 2008).  The size and therefore importance to practice profits was also a critical part of ensuring the routinisation of pay for performance into every day practice. Almost all interviewees felt that the income attached to QOF was appropriate. Concern was voiced that if a smaller percentage (less than 10%) was attached to achievement, it would be insufficient to motivate practice teams to work as hard (Lester et al 2013).  Although participants emphasised the importance of traditional general practice values, such as holism and continuity, the majority felt that the 2004 changes had impacted on these values. Participants related that patients now experienced less continuity with their GPs. Participants highlighted two major contributing factors to this: the role of practice nurses in conducting the day-to-day QOF template work, and the impact of opting out of providing out-of-hours care. While the changes meant individual GPs had increased flexibility in terms of work–life balance, they often perceived that patient care had declined as a result: Cheraghi-Sohi et al 2012.  In spite of this evidence of a move towards a more biomedical approach, all of our participants claimed that they still were able to practice ‘holistic’ medicine.  Careful analysis of these claims to holism suggested that they rested upon the somewhat slippery and variable definitions of ‘holism’ that exist. Thus, we found claims to holism variously based upon: a metaphorical ‘protected space’ within the consultation; an ideal of complexity that claimed that doctors continued to treat ‘complex’ patients whilst their nursing colleagues dealt with routine QOF related work; and the ability of doctors to maintain an ‘overview’ of patient care, even if they were not personally involved. Checkland & Harrison 2010. |
|  | The defining goal of this value type is personal success through demonstrating  competence according to social standards. Competent performance is a requirement if individuals are to obtain resources for survival and if social interaction  and institutional functioning are to succeed. Achievement values are mentioned  in many sources (e.g., Maslow, 1959; Rokeach, 1973; Scott, 1965). As defined here, achievement values emphasize demonstrating competence in terms of prevailing cultural standards, thereby obtaining social approval.  This differs from  McClelland’s (McClelland, Atkinson, Clark, & Lowell, 1953) definition of  achievement motivation to meet internal standards of excellence. The latter is  more closely related to self-direction values (ambitious, successful, capable, and influential). | **Achievement** –  Personal success through demonstrating competence according to social standards.  Ambitious  Influential  Capable  Successful  Intelligent  Self-respect | Increase in the number of administrative staff, including those with responsibility for information technology (IT) (Checkland & Harrison 2010). This new managerial stratum actively worked to align their colleagues’ clinical activities to the wider organisational goals (Cheraghi-Sohi & Calnan 2013). Practices had made extensive alterations to their organization in terms of staff appointments, skill mix, computer coding and appointment systems in order to more actively identify and recall patients, agree practice wide protocols in accordance with the QOF, deliver standardized care as a team, and consistently code care processes (Maisey et al 2008).  Internal hierarchies developed with, for example, promoting three receptionists to form the ‘IT team’, who not only had higher status than their reception colleagues, but who were also involved at a very early stage in decision making about how to address new QOF targets.  Managers’ roles gained in importance, as they assumed responsibility for delivering the 500 points devoted to ‘managerial’ domains and for overseeing the achievement in the clinical domains. (Checkland & Harrison 2010).  Since 2004, practice nurses carry out far more consultations – an increase from 21% to 35% of general practice consultations, 76 and there have been changes to professional boundaries (NHS England 2018).  Many routine tasks have passed from GPs to practice nurses or health care assistants, and there has been reorganisation of care into chronic disease clinics. Practice staff to carry out the information technology tasks needed to collate data for QOF has also increased (NHS England 2018).  Another frequently reported problem was the need for greater consistency over the timing and extent of changes to the individual indicators and the overall QOF.  Almost all GPs and practice managers described a sense of decreased clinical autonomy and loss of professionalism. They also described a sense of micromanagement from above and frequently cited the late communication about changes to the wider QOF and year-on-year variability in the occurrence and timing of changes to indicators as politically motivated micromanagement that reduced their clinical autonomy and sense of professionalism. Lester et al 2013.  This inconsistency was seen by interviewees as working against routinisation, creating a sense of uncertainty that almost all felt could be improved through better communication between policymakers and front line practitioners, and an agreed timetable for changes. Biennial changes were most frequently favoured (Lester et al 2013)  Most doctors and nurses acknowledged that nurses had become the primary provider of health care for patients with chronic conditions (Campbell et al 2008). Although most doctors welcomed the added value of this role, some doctors believed they had become deskilled in areas previously seen as a core part of their role (Campbell et al 2008). These nurses perceived their increased autonomy, hard work, and chronic disease management roles as critical in achieving QOF targets (Campbell et al 2008).  For some, this increase in autonomy and role expansion was in response to the GPs’ needs for work to be conducted in the area of chronic disease management to meet QOF targets, rather than in response to nurses’ clinical interests (McGregor et al 2008).  Case findings often occurred within tightly structured and time-limited chronic illness reviews required to document QOF processes of care, and appeared to exacerbate existing discordance. This led to professionals disregarding attempts by patients to steer the consultation around to their own perceived needs (Alderson et al 2014; Maxwell et al 2013).  When asked about the case finding, most nurses felt it was difficult to switch from asking something that could be measured (such as weight, units of alcohol consumed) to something more subjective. The case-finding questions appeared out of place in the consultation that mainly involved measuring physical factors rather than mood-related problems (Alderson et al 2014). The introduction of recommended tools was reported by both nurses and GPs as replacing a more holistic discussion with patients. They described this more mechanistic process as ‘less professional, and disrupting the normal patient/professional interaction. Nurses felt that the scripted questions required more surrounding dialogue (Maxell et al 2013).  Patients were often not focused on and often did not understand the purpose of the review process and used the consultation as an opportunity to raise other problems. To manage this, professionals often interrupted patients or returned the consultation to its purpose, discounting clues that the patient had worries related to the chronic disease being reviewed or other illnesses. Alderson et al 2014). The context of chronic illness reviews was restrictive—in this case, an opportunity for direct, subject-specific case finding was missed because of the necessity to ask about and record other items. (Alderson et al 2014; Maxell et al 2013)  Most physicians believed that the quality targets had improved patient care by focusing attention on necessary clinical activities that might have been being neglected (Campbell et al 2008). Participants were supportive of the systematic approach to care provided by the ICT systems and viewed the overall aim of QOF as a mechanism for improving and/or standardising quality of care within practices (Cheraghi-Sohi & Calnan 2013).  Since 2004, QOF-related workload, which focused particularly on patients with long-term conditions, was described by interviewees as becoming more structured and proactively planned and managed on an annual basis, supported by increased computerisation. Patients followed a predetermined pathway with evidence-based indicators marking progress at regular intervals and clinics often planned around prevalence of illness in a practice (Lester et al 2013).  The size and therefore importance to practice profits was also a critical part of ensuring the routinisation of pay for performance into every day practice. Almost all interviewees felt that the income attached to QOF was appropriate. Concern was voiced that if a smaller percentage (less than 10%) was attached to achievement, it would be insufficient to motivate practice teams to work as hard (Lester et al 2013).  Role of monitoring compliance with the coding regime which feeds into the contract monitoring system and of highlighting deficient coding and recording performance amongst staff, has contributed positively to this doctor’s sense of self-worth (McDonald Harrison Checkland 2008). Most salaried GPs however spoke of regularly meeting their targets for a variety of reasons. The two main reasons cited, in addition to the evidence-based nature of the majority of targets, were that; most eventually wished to become a partner in their practice and many had trained in their current practices, becoming socialized into their practice’s way of doing things, including meeting ‘their share‘ of QOF targets (Cheraghi-Sohi & Calnan 2013).  In order to direct their colleagues work to areas in deficit, QOF leads performed regular monitoring, most frequently, at weekly practice meetings. A variety of approaches were described such as ‘naming and shaming’ to motivate activity and improve performance. Ultimately, it appeared that many ‘non-chasers’ were willing to accept scrutiny by QOF leads, and even the shame of being ‘publicly’ chastised to avoid the future possibility of being seen as the deviant case or ‘outsider’ within their organisations (Cheraghi-Sohi & Calnan 2013). While such close scrutiny could have been seen as negative and constraining, they perceived this as being positive both for their own continuing development and for patient care (Cheraghi-Sohi & Calnan 2013).  Pay-for-performance schemes to retain, attract and motivate primary healthcare professional represent a narrow view of motivational and satisfaction mechanisms. An evidence synthesis on GP recruitment and retention show that there is little evidence to support the idea that financial schemes increase satisfaction or reduce demotivation or dissatisfaction.78 The evidence suggests that QOF may have positive and negative effects on motivation for primary care professionals and this highlights the need to consider both intrinsic and extrinsic motivational factors, and among extrinsic factors, to consider non-financial rewards (NHS England 2018). |
|  | Power values are probably grounded in more than one type of universal requirement. The functioning of social institutions apparently requires some degree  of status differentiation (Durkheim, 189311964; Parsons, 1957), and a dominance/submission dimension emerges in most empirical analyses of interpersonal relations both within and across cultures (Lonner, 1980).  To Justify this fact of  social life, and to motivate group members to accept it, groups must treat power as a value. Power values may also be transformations of the individual needs for  dominance and control identified by analysts of social motives (e.g., Korman,  1974; Schutz, 1958).  Power values have been mentioned by value analysts as well (Allport, 1961; Gordon, 1960). We view the central goal of power values as attainment of social status and prestige, and control or dominance over people and resources (authority, wealth, social power, preserving my public image, social recognition).  Both power values and achievement values focus on social esteem.  However,  achievement values (e.g., successful, ambitious) emphasize the active demonstration of competence in concrete interaction, whereas power values (e.g.,  authority, wealth) emphasize the attainment or preservation of a dominant position within the more general social system. | **Power** –  Social status and prestige, control or dominance over people and resources.  Social power  Wealth  Authority  Preserving public image  Social recognition | All the practices that were studied had changed their modes of operation in response to the QOF (Checkland & Harrison 2010). Practices appeared to have undergone a form of convergent evolution, or isomorphism, as all had developed QOF teams whose primary purpose was the routine monitoring and surveillance of their colleagues’ QOF-related work (Cheraghi-Sohi & Calnan 2013).  There had been substantial changes in practice organization and individual responsibilities, these included alterations in hierarchy, with nurses feeling empowered and teamwork in the practices being stronger and more coherent (Maisey et al 2008).  Clinical indicators were issued in April 2006, rather than being discussed initially by the doctors in order to discuss their clinical merits/demerits, the first meetings were held between the partner with QOF responsibilities and the IT team. Only once this group had produced an implementation plan was there any discussion with the wider team. In this way new indicators were configured as a technical problem requiring an IT solution, rather than as a clinical problem requiring a clinical response by the doctors (Checkland & Harrison 2010).  Having responsibility for checking the IT system to look for patients not meeting QOF targets gave the nurses (and doctors) concerned the legitimacy to ‘chase’ their colleagues by, for example, sending notes to request that certain checks were made when patients attended for routine consultations Checkland & Harrison 2010.  Professionals appeared more preoccupied by their lack of control in achieving indicator targets, especially if dependent upon patient cooperation (Hacket et al 2014). Limited availability of appropriate, supportive resources needed to address such problems further undermined confidence in these targets (Hacket et al 2014).  While the need for evolving content was generally accepted and perceived as legitimate if aligned to changing evidence, many changes were viewed purely in political terms. The legitimacy of the negotiators and architects of QOF was questioned: Cheraghi-Sohi & Calnan 2013  Although the new contract is a practice contract, and targets had been achieved by teams of doctors, nurses, and administrative staff, most of additional money became part of the doctors’ income as employers and owners of practices, regardless of the nurses’ contribution to achieving the targets (Campbell et al 2008). Roles and incentives were discussed in relation to two issues: professional development and professional status.  Workload had increased for all nurses in terms of clinical commitment (particularly in contract-driven chronic disease management and preventive care), bureaucracy, and data collection (McGregor et al 2008). Even those nurses who felt that the type of patient they were seeing hadn’t changed complained that the volume of work had increased dramatically. Most felt pressurised by this, with time being a particular constraint (McGregor et al 2008).  Although most nurses felt that their professional status had improved, many felt there were few tangible benefits in terms of salary, (McGregor et al 2008). One practice nurse was aware of the business side of the practice and felt that, although she received a monetary bonus, it was diminutive in comparison to the money awarded to the practice for the QOF points attained (McGregor et al 2008). No nurse had been offered the possibility of becoming a partner, despite this being allowed under the new contract. However, it was unclear whether this was due to GPs not offering nurses such opportunities or to the nurses themselves feeling reluctant to grasp them (McGregor et al 2008). Indeed, when some nurses were explicitly asked about nurse partnerships, most did not want to take on that level of responsibility, at least at that time (McGregor et al 2008).  Practices often offered practice nurses incentives at the end of the contract year, in the form of a monetary bonus or extra holidays. Opinion as to the appropriateness of the incentive varied, with no obvious association between QOF points and the incentives offered (McGregor et al 2008). One practice nurse was aware of the business side of the practice and felt that, although she received a monetary bonus, it was diminutive in comparison to the money awarded to the practice for the QOF point’s attained (McGregor et al 2008). The scheme augmented perceptions of unfair distributions of workloads and remuneration within practices, particularly between nursing and medical staff. Some nurses were keen to emphasize that they did not think that they should receive additional money for doing their job (Hacket et al 2014). However, several nurses were openly critical of the fact that whilst they did most of the work, it was the GPs who benefitted financially (Hacket et al 2014).  However, accepting personal responsibility for achieving targets and engaging in self-surveillance also placed nurses under pressure (McDonald Harrison Checkland 2008).  Whilst GPs chased medical colleagues, the Practice Manager “chased” other staff members, in a way, which risked undermining the motivational effects and self-surveillance of the nurse leads system (McDonald Harrison Checkland 2008).  In addition, nurses appeared to feel demotivated by what they perceived as the constant surveillance and criticism of their performance (McDonald Harrison Checkland 2008). In many cases, the changes appeared to result in lower tiers (PNs and HCAs) being allocated responsibility for major areas of routine QOF work. All participants spoke of internal QOF teams/leads whose remit was to actively pursue targets, monitor and direct other clinicians to ensure overall practice performance was raised and/or maintained (Cheraghi-Sohi & Calnan 2013).  Most doctors and nurses acknowledged that nurses had become the primary provider of health care for patients with chronic conditions (Campbell et al 2008). Although most doctors welcomed the added value of this role, some doctors believed they had become deskilled in areas previously seen as a core part of their role (Campbell et al 2008). Such situations contributed to a general feeling of inequity in the workload/remuneration balance between the nurses and GPs. Indeed, occasionally, the use of an inappropriate incentive was perceived to be worse (McGregor et al 2008)  Professional beliefs and abilities affected how case finding was undertaken. Alderson et al 2014. We noticed that those who felt that the case finding was for the benefit of patients appeared to work in practices that were in areas of low deprivation, whereas those in areas of higher deprivation felt there was a lack of time to ask the questions and deal with any responses that might indicate a problem with mood. In the context of a time restricted consultation, they felt overburdened. Alderson et al 2014. Professionals at nearly every practice mentioned the term ‘can of worms’ to express unease with case finding for depression ( Alderson et al 2014).  This metaphor indicated professional perceptions of both patient discomfort with being asked about emotions and their own emotional labour in asking the questions (Alderson et al 2014). Cans of worms helped articulate the belief that case finding for depression was anticipated as a problematic part of the consultation and threatened to derail routines (Alderson et al 2014). Nurses also reported concerns about a lack of services or options available if people were identified as depressed. This suggests a lack of knowledge or confidence for both GPs and Nurses concerning the availability of resources to help manage depressed patients (Maxell et al 2013). Nevertheless, despite similar reservations, one GP commented that having the two questions built into annual reviews ensured that screening for depression was not forgotten (Maxell et al 2013).  The vast majority of participants reported that they complied with all areas of the QOF, regardless of their personal opinions. Various reasons were cited for this, including pressure from internal surveillance, direct responsibility as part of the practice QOF team, contractual obligation, and trainee loyalty to the practice, and finally their desire to eventually become a partner in the practice (Cheraghi-Sohi et al 2012).  All participants acknowledged the sizeable pay differentials between themselves and their principals that had arisen due to the contractual changes. A small number had received QOF-related financial bonuses in the early stages of the contract at least, but these had since ceased. Whereas some were resentful of the differences, others felt that the inequality fairly reflected the contrasting levels of responsibility (Cheraghi-Sohi et al 2012). Despite their status as employees, most felt that they were fairly treated and highlighted examples such as their regular inclusion in practice meetings and the variety in the types of work that they were able to undertake. However, they also highlighted that this was somewhat context dependent (Cheraghi-Sohi et al 2012).  The universally high QOF achievement means that practices have little motivation to improve achievement further for existing indicators. Raising thresholds further may even lead to increased exception reporting in order to raise apparent achievement with no real increase in the desired activity. This means that QOF is unlikely to be an effective mechanism for improving performance, although the evidence suggests that it motivates practices to maintain performance – but as measured by QOF indicators alone (NHS England 2018). |
|  |  |  |  |
| **Openness to change** | The defining goal of this value type is independent thought and action choosing, creating, exploring.  Self-direction was derived from organismic needs for control and mastery (e.g., Bandura, 1977; Deci, 1975; White, 1959) and interactional requirements of autonomy and independence (e.g., Kluckhohn, 1951; Kohn & Schooler, 1983; Morris, 1956) (creativity, freedom, choosing own goals, curious, independent). | **Self-direction** -  Independent thought and action—choosing, creating, exploring.  Freedom  Creativity  Independent  Choosing own goals  curious  Self-respect | Previously, the practice had assumed that it was performing well, since complaints were low, patients appeared happy and staff was given a large degree of freedom when deciding on the appropriate way to deliver services. McDonald Harrison Checkland 2008  Professionals appeared more preoccupied by their lack of control in achieving indicator targets, especially if dependent upon patient cooperation (Hacket et al 2014). Limited availability of appropriate, supportive resources needed to address such problems further undermined confidence in these targets (Hacket et al 2014).  A ‘tick box’ approach to medicine encouraged by pay for performance indicators was also seen by a small minority of GPs and practice nurses as a further reduction in clinical autonomy.  The ‘black and white’ nature of indicators was seen as an inevitable consequence of their evidence-based nature, but also as something that caused a tension with the essentially ‘grey’ ambiguous nature of work in primary care (Lester et al 2013). The clinician interviewees described wanting to regain some control over their clinical work through modifying indicators to meet the needs of individual patients. These strategies included in the context of existing QOF, variable practice in using depression assessment schedules Lester et al 2013).  However, this created differences in how reviews and structured tools were implemented between clinicians and therefore the care received by patients in different practices (Lester et al 2013; Arbaje et al 2014).  In addition to surveillance of others, lead clinicians must also police their own behaviour in order to ensure their performance is beyond reproach (McDonald Harrison Checkland 2008). Whilst “chasing” nurses who are practice employees may be relatively easy, exerting authority over clinically autonomous GP partners may be more difficult. GPs who were not “chasers” waited until they were “chased”, rather than proactively pursuing contract targets (McDonald Harrison Checkland 2008). However, whilst non-chaser GPs were happy with this state of affairs, amongst those GPs with direct responsibility for QOF targets, discontent was apparent amongst clinicians with direct responsibility for QOF targets with colleagues perceived as “free riders” (McDonald Harrison Checkland 2008).  Doctors reported a more intensive working pattern as team leaders supervising the work of nurses (Maisey et al 2008). It was also apparent from their accounts that participants’ attitudes to their salaried status were dependent upon their personal aspirations. For example, a small number (n = 3) had actively rejected their prior principal status, trading off higher income for less responsibility and more time to pursue other careers. Others held no current desire for a partnership and wanted lower levels of responsibility due to family commitments, or they simply wished to concentrate and build up their clinical knowledge (Cheraghi-Sohi et al 2012).  Most participants, however, did eventually wish to become a partner, in order to have more control over practice affairs (Cheraghi-Sohi et al 2012). All participants were aware of the lack of availability of partnerships. For those who had actively chosen a salaried post, this was of little personal concern. Those who desired partnership status were mostly content to serve a period of ‘apprenticeship’, but held concerns over its duration. While many were hopeful that the situation was temporary, they were concerned as to long term effects on their career prospects, as well as professional morale (Cheraghi-Sohi et al 2012).  Focus on contract areas led several nurses to voice concerns at having fewer opportunities to work and train in non-contract areas, such as minor illness (McGregor et al 2008).  All doctors reported that there had been no change to the essence of the face-to-face doctor-patient interaction within routine consultations, which are booked at 10-minute intervals. Campbell et al 2008. Addressing a number of agendas within a single consultation was seen by many doctors as a key skill of good family practice and reflected a long-standing acknowledgment that a consultation could include acute and chronic problems, health promotion, and health prevention advice as appropriate to that consultation. Campbell et al 2008 |
|  | The motivational goal of stimulation values is excitement, novelty, and challenge in life (a varied life, an exciting life, daring).  Biologically based variations in the need for stimulation and arousal, conditioned by social experience,  may produce individual differences in the importance of stimulation values. Such a biological base has been claimed for thrill-seeking (Farley, 1986), a personality  variable related to stimulation values. | **Stimulation** -  Excitement, novelty and challenge in life.  An exciting life  A varied life  Daring | Most practice nurses felt they had expanded their role and taken on new skills, particularly in chronic disease management and data recording, since the implementation of the new GMS contract. This view was consistent across practices, regardless of the level of QOF achievement or the socioeconomic profile of the practice population: McGregor et al 2008  For nurses delegation of responsibility for targets appeared to be a source of motivation, with the “Population Manager” computer system providing feedback on performance against targets (McDonald Harrison Checkland 2008).  The introduction of the new contract meant that rather than trusting staff to deliver quality services, the practice moved to a system involving greater surveillance and scrutiny of performance. In addition to self-surveillance, with individuals monitoring their own performance, the availability of performance data also facilitated surveillance by other colleagues and the Practice Manager (McDonald Harrison Checkland 2008). However, GPs who were designated leads, rather than trusting to colleagues to hit targets, adopted an approach of surveillance and feedback, communicating areas of underperformance to medical colleagues (McDonald Harrison Checkland 2008). Surveillance and feedback on perceived deficiencies might improve performance in the short-term, however, it may have unintended consequences in the long-run, by conveying to staff that they are not trusted to deliver and reducing their motivation (McDonald Harrison Checkland 2008).  The scheme seemed to influence adherence to the targets primarily through motivational means, supported by other mechanisms. Motivations were extrinsically and intrinsically driven (Hacket et al 2014).  Professionals from practices serving both affluent and deprived populations felt the scheme legitimised their intrinsic motivation to improve patient outcomes (Hacket et al 2014). Others, particularly practices serving more deprived populations, appeared to be directly amenable to financial reward as an extrinsic driver. However, there were concerns that financial rewards from the scheme may not have been worth the effort involved in achieving targets and that the scheme did not directly target most of the people actually doing this additional work (Hacket et al 2014).  For practice managers and GPs in affluent high performing practices, competition and implicit threats to status also emerged as motivators (Hacket et al 2014). There were three other ways in which the scheme appeared to influence clinical behaviour. Firstly, several high performing practices and one low-performer had adapted templates provided by the PCT to support processes of care and recording in consultations. Practitioners from these practices considered that such prompts had been helpful (Hacket et al 2014). Secondly, some health professionals and developers of the scheme felt that it promoted standardised care and believed that adherence to the indicators had become routine practice. Consultation templates supported this setting of new norms within clinical routines (Hacket et al 2014). Thirdly, the social influence of having a member of practice staff as the champion for the scheme promoted engagement (Hacket et al 2014). |
|  |  |  |  |
| **Self-transcendence** | This is a more narrowly defined version of the earlier prosocial value type.  Whereas prosocial referred to concern for the welfare of all people in all settings, benevolence focuses on concern for the welfare of close others in everyday  interaction. The narrow focus is more in keeping with Schwartz and Bilsky’s  (1987) derivation of this type from the need for positive interaction in order to promote the flourishing of groups (cf. Kluckhohn, 1951; Williams, 1968) and  from the organismic need for affiliation (cf. Korman, 1974; Maslow, 1959).  The motivational goal of benevolence values is preservation and enhancement of the  welfare of people with whom one is in frequent personal contact (helpful, loyal, forgiving, honest, responsible, true friendship, mature love). | **Benevolence** -  Preserving and enhancing the welfare of those with whom one is in frequent personal contact (the ‘in-group’).  Helpful  Responsible  Forgiving  Honest  Loyal  Mature love  True friendship | (Changes resulting from introduction of the QOF on practice organisation, leading to an increased role for information technology) (Checkland K, Harrison 2010),  Although many doctors and nurses believed the templates played a positive role in prompting them to take action, such as measuring blood pressure, others believed the templates introduced a new element into the consultation and threatened to shift the balance away from the patient’s immediate agenda (Campbell et al 2008).  While better information technology was an important component of improvements in care, there was a downside to increased computer use in the consultation (Maisey et al 2008).  Prompts on the computer screen, though seen as useful in ensuring coverage of appropriate clinical activities, were identified by most respondents as a significant distraction from the patient’s concerns (Maisey et al 2008).  Thus, for example, who is allowed to enter data into a record is both shaped by and will shape the organisational hierarchy. Similarly, a record that only allows the recording of ‘yes/no’ factual data that can be coded into categories will tend to crowd out and devalue softer, more nuanced contextual information. (Checkland & Harrison 2010).  A significant minority of GPs also stated that they were more likely to exception report indicators with poorly perceived face validity. This was because the care/treatment element of the indicator was felt to be contrary to their role as the patient’s advocate and, in their clinical judgement, not relevant to individual patient-centred care (Campbell, Hannon, Lester, 2011). Almost all interviewees emphasised the value of piloting in terms of an opportunity to identify unintended consequences of potential QOF indicators in ‘real world’ settings with staff who deliver day-to-day care to patients. This enabled potential problems arising from implementation to be identified and addressed prior to the indicator being used. Lester, Hannon, Campbell 2011.  Doctors were less satisfied overall, as their continuity with individual patients had tended to decrease, while managerial and supervisory roles were enhanced (Maisey et al 2008).  Unlike the doctors who were able to claim that the contract did not hamper their ability to deliver patient-centred care, nurses expressed concerns about the target regime and its potentially negative consequences for patient-centred care.  Only two GPs reported that they had collectively decided not to pursue certain areas of QOF as they did not consider them to be ‘evidence based’ as a result they were prepared to trade-off this portion of their income, in order to continue to practice in a manner that was consistent with their personal and professional values (Cheraghi-Sohi & Calnan 2013).  In terms of autonomy and holism, some participants reported misgivings with regard to particular aspects of the QOF, particularly in areas where they questioned the evidence base for some of the targets. Concerns were raised about the prioritisation of incentivised care and the loss of holism (Cheraghi-Sohi et al 2012). Several GPs discussed the complexity of primary care particularly in areas of high social deprivation and with large immigrant populations. Participants stated that if markers are linked to payment yet failed to capture the clinical context and the challenges of general practice there may be unintended consequences (Gill et al 2012).  Respondents described greatly increased activity around the performance and recording of the incentivized indicators, with most interviewees emphasizing increased clinical activity, rather than improved recording methods alone (Maisey et al 2008). Most perceived this as better quality of care overall, nurses in particular saw greater consistency of care as an important improvement, but many respondents felt the focus on ‘QOF’ indicators compromised patient-centeredness, continuity, and patients’ choice of service (Maisey et al 2008).  Whereas most doctors believed that they treated all patients and patient conditions equally, a minority wondered whether there had been a subtle downgrading of conditions not incentivized in the QOF, as well as the social aspect of family medicine, through changes to available education and increased time pressures within consultations (Campbell et al 2008). There was no consensus about whether addressing targeted areas had led to neglecting those areas within the consultation that are not incentivized (Campbell et al 2008).  Some doctors commented on how pursuing QOF targets within a consultation could create a potentially conflicting doctor and patient dual agenda (Campbell et al 2008 Hacket et al 2014). Difficulties arose in the consultation when the patient mentioned a problem that the health professional perceived to be important but unrelated to the disease under review. Alderson et al 2014). There were also concerns about adding more and more into consultations. Patients with, for example, serious mental illness were described as often consulting opportunistically, rather than at specific appointment times. The problem of time for the consultation and screening extended to the problem of dealing with a positive result; with concerns that the clinician might be overwhelmed by opening a ‘Pandora's box’ or ‘can of worms’. As a result, questions may be asked in a way which discouraged the patient to respond (Maxell et al 2013).  Where QOF was not able to be completed, due to complex consultations or if GPs felt it ‘inappropriate’ to raise QOF, patients were asked to re-attend Cheraghi-Sohi & Calnan 2013). Participants were confident however that these patients or QOF points would not be missed due to the call and re-call systems Cheraghi-Sohi & Calnan 2013 Checkland & Harrison 2010). Sometimes the review had to be abandoned as the patient’s agenda became too important to be ignored, or the patient too distressed to continue concentrating on the review. Alderson et al 2014.  Others described incorporating the QOF agenda while attempting to deal with the patient’s agenda, despite having reservations about the value of QOF work, whereas still others stated that this double agenda added to the workload of consultations and risked doctors being distracted from the patient’s agenda. Campbell et al 2008).  The patient’s presentation indicates possible memory problems, which the GP does not address whilst conducting the review, instead bypassing them to focus on the QOF template of blood and urine tests. Although the GP seemed aware that the patient was having difficulty understanding how to organize blood tests, he dealt with this confusion by directing the patient to the reception staff, rather than explore possible memory problems. The patient and his wife seem superficially engaged in the process of review, but later describe feeling that the review had added nothing to his care, which remains fragmented, with problems unaddressed. Fragmentation is observable in other aspects of the patient’s care beyond the review, despite the practitioner arguing for the review’s essential role in providing “holistic” care. Chew-Graham et al 2013  Moreover, participants acknowledged that their consultations had become more ‘biomedical’, with an additional QOF-related agenda running alongside the patient’s own agenda. Checkland & Harrison 2010.  The context and consequences of the doctor patient interaction, however, were perceived by all doctors as having changed as a direct result of the pay-for-performance scheme. All participants acknowledged that the QOF had influenced their agenda. Campbell et al 2008 Cheraghi-Sohi & Calnan 2013. QOF could influence the process and structure of the consultation; it also appeared that GPs held wide discretion in whether and how to approach QOF, as individual approaches to attending to QOF in consultations varied. Cheraghi-Sohi & Calnan 2013. GPs still perceived that they still held wide ‘task discretion’ within their consultations Cheraghi-Sohi & Calnan 2013.  For most nurses, the inclusion of questions on emotional health at the end of a long list of physical health priorities minimised its importance. The resultant manner in which the questions were administered discouraged patients from disclosing any problems. Maxell et al 2013.  Although participants were supportive of QOF due to it being largely evidence-based, they were keen to point out that such rules were not simply mechanistically applied to all patients, but that there was a continued need to interpret them for each individual patient Cheraghi-Sohi & Calnan 2013.  All were keen, however, to emphasize that the patient’s agenda came first and that QOF reminders flashed up on the computer would be bypassed if there was in sufficient time to address both. Campbell et al 2008  Patient-centred approach appeared to occur more often in practices that had a lower than average QOF achievement, suggesting that such practices traded off potential income against responsiveness to patients. Alderson et al 2014  There was surprisingly little discussion of the importance of patient concerns as a measure of practice performance. Maisey et al 2008  The use of patient surveys to improve practice quality, which is also incentivized in the payment scheme, was not perceived as a driving force: respondents displayed only vague recall of survey activity or impact on practice services, despite a specific question prompt. The surveys were seen as ‘political correctness’, without the same evidence base that underpinned the clinical standards. Maisey et al 2008  Participants felt that they already knew their patients’ views and had attempted to meet their expectations as far as was practical, but that these expectations were often unrealistic, unachievable and contrary to the terms of the contract. Maisey et al 2008  The exception to this was appointment systems, which several practices had changed in response to problems identified by patients in the patient survey – for example, by putting in extra phone lines and staff at peak times. Maisey et al 2008  These exception codes were often entered onto to the practice system by employed non-clinical practice staff. Given that not all indicators were equally relevant to the needs of each individual patient on a given register, the second underlying principle related to an assertion that exception reporting was an integral part of the clinical judgment required in implementing indicators. This use of clinical judgment was expressed in two ways, one of which was through the use of specific ‘discretionary’ exception rules, such as patients being on maximal tolerated therapy. Participants also spoke of how exception reporting allowed them to focus on an individual patient’s circumstances, contexts, or choices; for example, where a patient was old and frail or had multiple morbidities. This use of discretionary clinical judgment exception codes was always decided on, and almost always entered, by GPs. Campbell, Hannon, Lester, 2011  One family doctor spoke specifically about how unfair it would be to remove exception reporting from practices in areas of deprivation, and another made the same point with regard to patients who often don’t attend when specifically invited to. Campbell, Hannon, Lester, 2011. |
|  | The motivational goal of universalism is understanding, appreciation, tolerance, and protection for the welfare of all people and for nature.  This contrasts  with the narrower focus of benevolence values.  The motivational goal of universalism values can be derived from those survival needs of groups and individuals  that become apparent when people come into contact with those outside the extended primary group and become aware of the scarcity of natural resources.  People may then realize that failure to accept others who are different and treat them justly will lead to life-threatening strife, and failure to protect the natural  environment will lead to the destruction of the resources on which life depends. | **Universalism** –  Understanding, appreciation, tolerance, and protection for the welfare of all people and for nature.  Equality  Unity with nature  Wisdom  A world of beauty  Social justice  Broad-minded  Protecting the environment  Peace | These concerns are similar to what other participants described about difficulties defining quality and being held accountable for measures they feel they cannot impact, such as patient nonadherence to their medical regimen, unexpected events after discharge, and dependence on more than one health care professional to ensure smooth transitions (Arbaje et al 2014).  Since deprivation affects both health and healthcare, differing deprivation of practice areas led to diverse interpretations of the indicators, particularly in the context of the physical health-check indicators for people with serious mental illness. Practices in particularly deprived areas were likely to have more patients with serious mental illness registered than those in less deprived areas. Such practices were far more likely to comment about the difficulty of engaging with patients with psychosis to carry out the annual check-ups. But mental health patients are particularly difficult because they’re not the sort of patients you can say come back next week. Lester, Hannon, Campbell 2011.  Some nurses felt that there was more of a focus on population health than on the needs of the individual. They were particularly frustrated by the rigid protocols for reviewing patients and the call and recall system (McGregor et al 2008).  Doctors, especially, expressed concerns about loss of holistic care and a skewing of effort towards incentivized activities. Maisey et al 2008  There was, however, an interesting tension for a considerable minority of doctors. These physicians described how the generally accepted desire to meet target levels and provide better chronic disease management also meant that at times they felt they might be too proactive in following up patients to meet targets or attend appointments, reflecting a physician-centred, rather than a patient-centred, approach to care. Campbell et al 2008.  If you ask [providers] who is responsible for that patient the moment they walk out of the door, you’d get a variety of answers. Some doctors, hospitalists, and residents believe it was them, even after their primary care doc saw them. But I bet a significant number would say when they’re out that door, “I’m done and I don’t have to worry about it anymore,” which is not true. And I think if you ask primary care docs, “Who’s responsible for this patient after discharge until they come see you?” a minority would say it would be the primary care doc and most would say it’s the hospitalist or the resident. Arbaje et al 2014. This statement reflects the physician’s concern related to confusion about responsibility for the care of the patient, which can potentially leading to a circumstance in which no health care professional is accountable for the patient. Arbaje et al 2014.  We found no evidence that QOF encourages any other aspect of primary care performance than those elements incentivised by the QOF. In particular, it does not reward holistic care, integrated care or patient-centred care.  Therefore, there is no evidence that QOF will advance progress towards the aims of the Five Year Forward View significantly. QOF encourages a narrow, biomedical view of health care performance, and ‘high performance’ does not necessarily mean ‘high quality’. The evidence suggests that QOF may divert practices and professionals from ways of providing high 32 quality of primary care that are not QOF-related. QOF does not incentivise practices to target patients with the greatest needs for primary care because these are more likely to be accepted.  Even if they are not accepted, practices have no motivation to prioritise more difficult-to-treat patients over those with less complex problems. We found no evidence that QOF is an effective mechanism for reducing inequalities in health and health care. It may even worsen inequalities if patients in whom clinical objectives are more easily met are targeted by QOF activities rather than those with more complex health and social problems (NHS England 2018).  We found no definitive evidence that QOF has any significant effect on neither population health nor emergency admissions. We also found no evidence of its cost-effectiveness, so its value cannot be compared with that of other health care interventions. We found no definitive evidence to inform us what would happen to performance on QOF indicators were the financial incentive removed, and no evidence to inform us what would happen to quality of primary care – although it is unlikely that this would be significant given the narrow view of quality that the QOF embodies (NHS England 2018). |
